# Processes independent of nonphotochemical quenching protect a high-light-tolerant desert alga from oxidative stress

**DOI:** 10.1093/plphys/kiae608

**Published:** 2024-11-09

**Authors:** Guy Levin, Michael Yasmin, Oded Liran, Rawad Hanna, Oded Kleifeld, Guy Horev, Francis-André Wollman, Gadi Schuster, Wojciech J Nawrocki

**Affiliations:** Faculty of Biology, Technion, Haifa 32000, Israel; Faculty of Biology, Technion, Haifa 32000, Israel; Kineret Limnological Laboratory, Israel Oceanographic and Limnological Research, Migdal 14950, Israel; Faculty of Biology, Technion, Haifa 32000, Israel; Faculty of Biology, Technion, Haifa 32000, Israel; Department of Biotechnology, Migal Galilee Research Institute Ltd, Qiryat Shemona 11016, Israel; Unité Mixte de Recherche 7141, Centre National de la Recherche Scientifique, Sorbonne Université, Institut de Biologie Physico-Chimique, Paris 75005, France; Faculty of Biology, Technion, Haifa 32000, Israel; Grand Technion Energy Program, Technion, Haifa 32000, Israel; Unité Mixte de Recherche 7141, Centre National de la Recherche Scientifique, Sorbonne Université, Institut de Biologie Physico-Chimique, Paris 75005, France

## Abstract

Nonphotochemical quenching (NPQ) mechanisms are crucial for protecting photosynthesis from photoinhibition in plants, algae, and cyanobacteria, and their modulation is a long-standing goal for improving photosynthesis and crop yields. The current work demonstrates that *Chlorella ohadii*, a green microalga that thrives in the desert under high light intensities that are fatal to many photosynthetic organisms does not perform nor require NPQ to protect photosynthesis under constant high light. Instead of dissipating excess energy, it minimizes its uptake by eliminating the photosynthetic antenna of photosystem II. In addition, it accumulates antioxidants that neutralize harmful reactive oxygen species (ROS) and increases cyclic electron flow around PSI. These NPQ-independent responses proved efficient in preventing ROS accumulation and reducing oxidative damage to proteins in high-light-grown cells.

## Teaser

*Chlorella ohadii* thrives under extreme light intensities using a unique photoprotective mechanism independent of nonphotochemical quenching.

## Introduction

Light drives photosynthesis but also damages the photosynthetic apparatus in a number of ways. Particularly, illumination facilitates the generation of reactive oxygen species (ROS) which damage the photosynthetic proteins, thereby inhibiting the process—an effect known as photoinhibition (Ögren 1988; Ögren and Sjöström 1990). Two major harmful photosynthesis-induced ROS are singlet oxygen (^1^O_2_) and superoxide (O_2_^•−^), and to detoxify them, photosynthetic organisms use distinct mechanisms. While O_2_^•−^ can be neutralized in a series of enzymatic-mediated reactions, ^1^O_2_ is chemically scavenged. Several molecules are known ^1^O_2_ quenchers, including carotenoids, tocopherols, fatty acids, and ascorbate (for recent reviews, see Triantaphylidès and Havaux (2009), Choudhury et al. (2017), and Hasanuzzaman et al. (2020)). Additionally, plants, algae, and cyanobacteria utilize various mechanisms to prevent ROS generation in the first place by quenching chlorophyll excited states before the energy can be transferred to oxygen. Under excess light conditions, nonphotochemical quenching (NPQ) mechanisms through which excess absorbed light energy is harmlessly dissipated as heat become critical for photosynthesis protection (photoprotection) (Delosme 1967; Wolff and Witt 1969; Demmig-Adams et al. 1989, 1990). The major NPQ component, q_E_, is located within the membrane-intrinsic major light-harvesting complex (LHCII) for photosystem II (PSII) and is activated by the acidification of the lumen compartment which results from photosynthetic electron transport. Typically, q_E_ is the strongest and the most rapidly triggered component of NPQ in the green lineage, undergoing activation in seconds, as recently reviewed by Bassi and Dall’Osto (2021). During periods of high photosynthetic activity, the thylakoid lumen is acidified due to proton translocation across the thylakoid membrane by the combined activity of linear and cyclic photosynthetic electron flows (LEF and CEF). Photosystem II subunit S (PsbS) and the light-harvesting complex stress-related (LhcSR) in plants and algae, respectively, act as proton sensors which, upon acidification of the lumen, induce q_E_ to dissipate excess energy as heat (Li et al. 2000, 2004; Peers et al. 2009; Liguori et al. 2013). Simultaneously, the xanthophyll cycle is activated, and newly formed zeaxanthin interacting in the PSII–LHCII chlorophyll pool provides pathways for energy dissipation (Demmig et al. 1987; Niyogi et al. 1998; Xu et al. 2015; Sacharz et al. 2017).

*Chlorella ohadii* is a green microalga that thrives in the desert, where it is exposed to extremely high light intensities (Treves et al. 2013; Levin et al. 2021). Accordingly, *C. ohadii* is highly tolerant to photoinhibition (Treves et al. 2016; Levin et al. 2023, 2024). The adaptation of *C. ohadii* to excess light involves marked changes in thylakoid content of which the most pronounced is a decrease in LHCII, as well as the massive accumulation of carotenoids and several photoprotective proteins (Levin et al. 2021, 2023). High-light (HL)-grown *C. ohadii* does not undergo state transitions (Levin et al. 2023), a mechanism of antenna exchange between PSII and PSI (Bonaventura and Myers 1969; Chow et al. 1981; Delosme et al. 1996), and a functional component of NPQ (q_T_) (Allorent et al. 2013; Nawrocki et al. 2016). Moreover, *C. ohadii* also lacks genes encoding LhcSR proteins nor does it accumulate the PsbS protein (Treves et al. 2016; Levin et al. 2023; Murik et al. 2024), suggesting the absence of q_E_. Interestingly, a carotenoid biosynthesis-related protein (CBR), which belongs to the LHC-like protein family with a structure similar to PsbS, accumulates to a great extent in HL-grown *C. ohadii* (Levy et al. 1993; Levin et al. 2023; Levin and Schuster 2023). Structural modeling suggested that one of the proton-sensing glutamates of PsbS was conserved in CBR, raising the possibility of its role in lumen acidification sensing. Moreover, fractionation of the photosynthetic protein complexes by sucrose density gradient centrifugation showed that CBR from HL-grown cells co-localized with a detached fraction of LHCII and carotenoids, both of which are associated with NPQ and ROS scavenging (Levin et al. 2023). Given that LHCII is the major site of NPQ (Ruban et al. 1999; Elrad et al. 2002; Sacharz et al. 2017; Nicol and Croce 2021), its elimination and the lack of LhcSR raise doubts as to the contribution of a q_E_ regulation which require to investigate further the mode of photoprotection in *C. ohadii*.

In this work, the contribution of the decrease in PSII antenna to HL acclimation and the interplay between NPQ and CEF on ROS accumulation and oxidative damage in *C. ohadii* are investigated. We demonstrate that q_E_ is actually absent and replaced with an NPQ-independent photoprotection in HL-grown *C. ohadii* cells. This mechanism, rather than allowing energy dissipation, mainly relies on limiting light absorption. It is concomitantly coupled to a dramatic enhancement of CEF around PSI and a vast accumulation of CBR, carotenoids, and other antioxidants, functionally reducing ROS accumulation. Thus, *C. ohadii,* adapted to grow at extremely high light intensity, employs both active and passive mechanisms of resistance to photoinhibition. The present work demonstrates that NPQ is not required to withstand excess illumination in HL-grown *C. ohadii* and that under conditions of prolonged exposure to HL intensities, minimizing the PSII–LHCII absorption cross-section and ROS production while maximizing antioxidant activity has been selected as the most successful strategy to reduce photoinhibition.

## Results

### HL-grown cells upregulate CBR and enzymatic antioxidant enzymes

**Figure 1.**
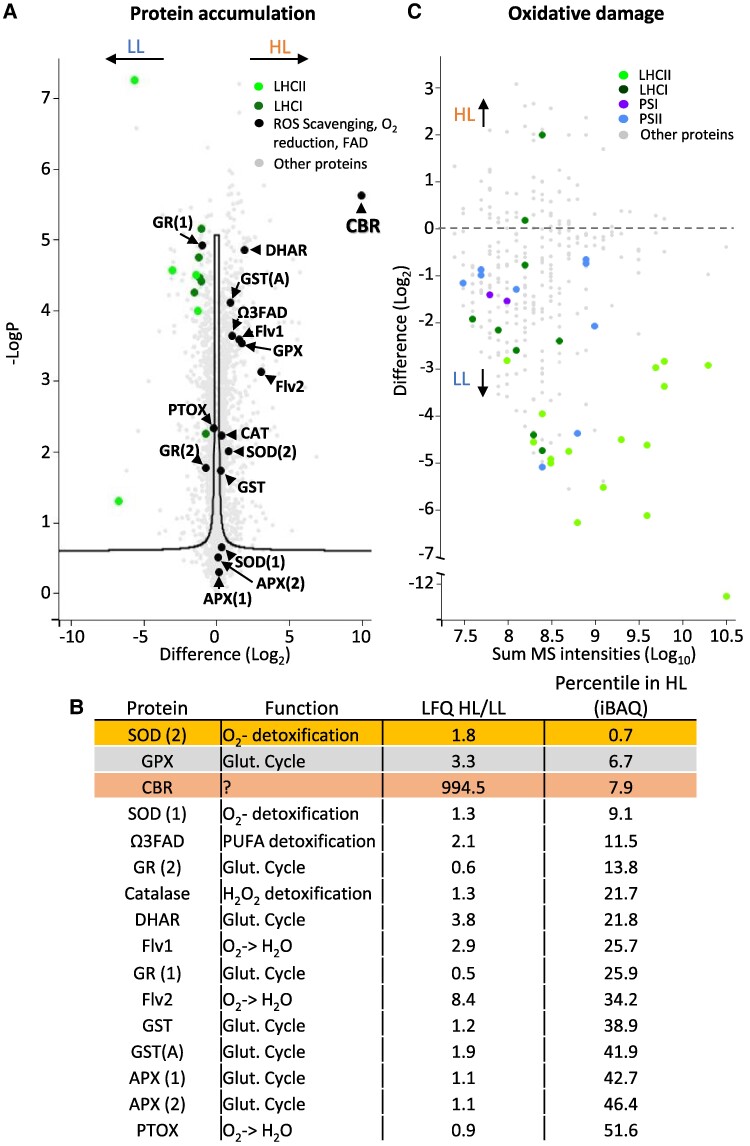
Acclimation to high-light conditions in *C. ohadii* as revealed by MS. **A)** LFQ liquid chromatography–tandem MS (LC–MS/MS) in LL- and HL-grown cells reveals the accumulation of known enzymatic antioxidants and CBR in HL-grown cells. Note the substantial reduction in LHCII subunits in HL-grown *C. ohadii*. The difference on the *x*-axis is between the mean LFQ intensity values for each protein from 3 biological repeats of each growth condition (LL and HL). The black line represents the statistical significance threshold, applying a permutation-based FDR of 0.05 and an S0 parameter of 0.1. **B)** List of the detected antioxidants and other related enzymes. Values in the “LFQ HL/LL” column represent the fold-change value of a given protein in HL over LL-grown cells, calculated as described above. Values in the “Percentile in HL (iBAQ)” column represent the abundance of the protein within HL-grown cells. A value of 10 means this protein is among the 10% most abundant proteins in these cells, based on its iBAQ value. **C)** Detected peptides with oxidized tryptophan as a mark for oxidative damage. Note the accumulation of almost all detected oxidized peptides in LL-grown cells. The difference on the *y*-axis is between the mean LFQ intensity values for each peptide with an oxidized tryptophan from 3 biological repeats of each growth condition. The sum of the intensities in both LL and HL growth conditions is displayed on the *x*-axis. SOD, GPX. CBR, Ω3FAD, GR, glutathione reductase; GST, glutathione S-transferase; PTOX, plastid/plastoquinol terminal oxidase.

*Chlorella ohadii* grows in biological soil crusts that form thin layers on the desert sand, where they are most often exposed to direct sunlight (Treves et al. 2013). To understand the remarkable resistance of *C. ohadii* to exposure to a high-light regime, we grew it for 24 h in heterotrophic conditions under low- and high-light conditions (LL: 50 µmol photons m^−2^ s^−1^; HL: 2,000 µmol photons m^−2^ s^−1^). These intensities mimic approximately the light intensities during the early morning and late evening, as well as during the cloudy winter days (LL), compared with a mid-summer day (HL). The cells were then subjected to label-free quantification mass spectrometry (LFQ-MS) to detect possible enzymatic antioxidants that participate in the detoxification of ROS and determine their effect on chloroplast protein oxidation. As was observed for isolated thylakoid membranes (Levin et al. 2023), the most prominent protein in HL-grown cells was CBR, with a 995-fold change compared with LL-grown cells. Although no enzyme is known to directly quench ^1^O_2_, CBR was previously reported to bind carotenoids (Levy et al. 1993), which are known as efficient ^1^O_2_ scavengers and strongly accumulate in HL-grown cells. In HL-grown *C. ohadii*, CBR was co-localized with the accumulated carotenoids (Levin et al. 2023). The CBR accumulation was accompanied by a drastic loss in LHCII (Fig. 1A). Changes in LHCI abundance were much smaller than in LHCII, which suggests that PSII light harvesting was more affected than PSI. Additionally, we identified an increase in 15 proteins associated with O_2_^•−^ detoxification or with energy dissipation via alternative electron transfer pathways (Fig. 1A and B). Specifically, we detected 2 isoforms of superoxide dismutase (SOD), which catalyzes the detoxification of superoxide (O_2_^•−^) by converting it to H_2_O_2_, one of which showing a 1.8 higher accumulation in HL-grown cells than in LL-grown cells. Of glutathione peroxidase (GPX) and ascorbate peroxidase (APX), which can further detoxify H_2_O_2_ by catalyzing its conversion to water (H_2_O), only GPX was significantly accumulated in the HL-grown cells (3.3-fold change). Dehydroascorbate reductase (DHAR) and 2 isoforms of glutathione S-transferase, which participate in detoxifying O_2_^•−^ via the ascorbate–glutathione cycle, were also significantly accumulated in HL-grown cells (3.8-, 1.9-, and 1.2-fold change, respectively). There were no statistically significant changes for catalase, which catalyzes the conversion of H_2_O_2_ to H_2_O in HL-grown cells. Although the accumulated enzymes reported here mainly contribute to O_2_^•−^ detoxification, their accumulation may indicate enhanced recycling of ascorbate, a known chemical scavenger of ^1^O_2_ (Triantaphylidès and Havaux 2009). ω-3 fatty acid desaturase (ω3FAD), which catalyzes the generation of polyunsaturated fatty acids (PUFAs), underwent a 2.1-fold increased accumulation in HL- compared with LL-grown cells. PUFAs have been suggested to contribute to ROS detoxification by interacting with ROS during lipid peroxidation (Schmid-Siegert et al. 2016). Flavodiiron proteins (Flv1 and Flv2), which act as electron sinks possibly involved in PSII and/or PSI photoprotection (Chaux et al. 2015; Burlacot et al. 2022; Bag et al. 2023), were significantly accumulated in HL-grown cells (2.9- and 8.4-fold change, respectively), providing an additional route for energy dissipation. In contrast, PTOX, another putative electron sink (Nawrocki et al. 2015), was not differentially accumulated in HL-grown cells (0.9-fold change) (Fig. 1A and B).

To further understand whether the marked change in abundance of detoxification enzymes did not merely result from their absence in LL, we relied on intensity-based absolute quantification (iBAQ) (Schwanhäusser et al. 2011) (Fig. 1B). Out of the abovementioned enzymes, SOD was the most abundant (top 0.7% percentile compared with all detected proteins) in HL-grown cells, followed by GPX (6.7%) and CBR (7.9%) (Fig. 1B). This further highlights the contribution of enzymatic antioxidants for photoprotection in HL-grown *C. ohadii* cells from O_2_^•−^, while CBR and the large accumulation of carotenoids protect from ^1^O_2_.

### HL-grown cells accumulate less oxidatively damaged proteins

To functionally assess the effects of ROS detoxification, the extent of oxidative damage was assessed by quantifying the irreversible oxidation of tryptophan—itself leading to protein degradation (Kato et al. 2023). Remarkably, there were more oxidized peptides from PSII, LHCII, PSI, and LHCI subunits, accumulating in LL-grown cells when compared with HL-grown cells (Fig. 1C), despite the 20-fold lower illumination intensity. This observation underlines the high efficiency of ROS scavenging in HL-grown cells. Taken together, our proteomics analysis suggests that SOD and GPX accumulate in response to HL growth conditions and play a major role in reducing O_2_^•−^-induced oxidative damage in HL-grown cells. Additionally, the marked accumulation of ^1^O_2_-quenching carotenoids (Levin et al. 2021, 2023), whether or not bound to CBR, should further reduce oxidative damage. Last, the robust downregulation of LHCII minimizes the uptake of excess light energy, thus reducing ROS generation. These changes result in a substantially lower accumulation of oxidized photosynthetic proteins in HL-grown cells.

### High-light acclimation results in LHCII loss and reduced PSII functional antenna

**Figure 2.**
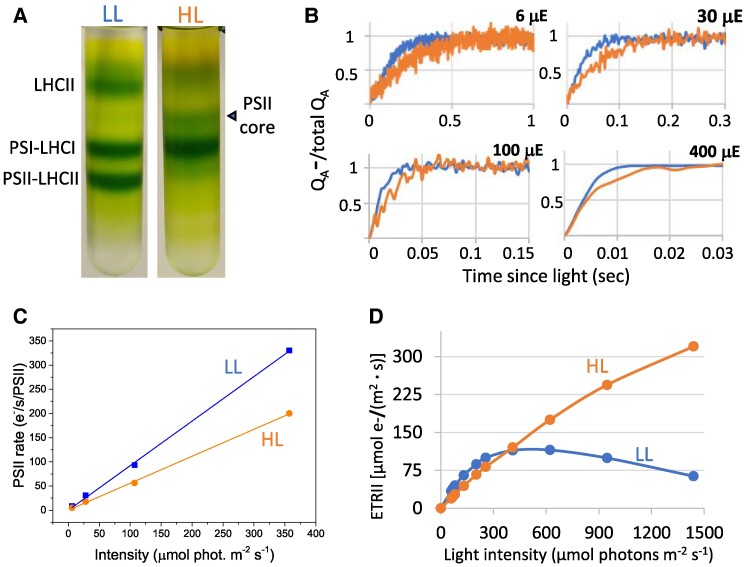
High-light-grown *C. ohadii* drastically decreases PSII antenna size. **A)** Thylakoid membranes from LL- and HL-grown cells were solubilized with detergents, and the photosynthetic complexes were separated by a sucrose density gradient. Samples containing the same amount of chlorophyll (200 µg) were loaded on the gradients. Note the accumulation of a PSII core, stripped from LHCII, in HL-grown cells. **B)** The kinetics of Q_A_ reduction upon exposure to actinic light in the presence of DCMU was measured with a fluorometer and directly corresponded to the PSII antenna size. Y(II) values (Φ_PSII_) were determined under the same experimental conditions, without adding DCMU. Fluorescence data were normalized to maximum (*F*_M_ = 1) and minimum (*F*_0_ = 0) values for each sample. The maximal photochemical rate of PSII at each light intensity was calculated by taking the reciprocal of the area delimited by *t*0, the fluorescence rise curve, and the value of 1 (i.e. *F*_M_) on the ordinate axis. μE refers to light intensity m^−2^ s^−1^. **C)** Comparison of the slopes of linear regression fits across the measured rate points indicates a ∼40% decrease in PSII absorption capacity in high-light-grown *C. ohadii*. **D)** Electron transport rates of PSII (ETRII) were normalized according to the calculated antenna size and tested under various light intensities. HL-grown *C. ohadii* ETRII reaches peak values at higher light intensities due to its truncated PSII antenna. All experiments are representative of at least 3 biological repeats.

To understand the extent of losses in LHCII complexes, thylakoid membranes isolated from *C. ohadii* grown under LL and HL conditions were solubilized and separated by centrifugation on sucrose density gradients. As shown in Fig. 2A, LL-grown cells displayed, in addition to a PSI–LHCI supercomplex, a PSII–LHCII supercomplex, and some free LHCII. In contrast, while we observed no changes in PSI–LHCI between cells grown at either light regime, we found no PSII–LHCII supercomplex in HL-grown *C. ohadii*. In contrast, only the PSII core complex was visible on the gradient.

To examine how the loss in LHCII and the accumulation of CBR could affect light harvesting in PSII, we performed measurements of its functional antenna size. To this end, we studied the fluorescence rise in the presence of the PSII inhibitor DCMU, which measures the Q_A_ reduction rate upon continuous illumination. The functional antenna size is approximated by calculating the reciprocal of the area above the fluorescence rise curve. A larger area relates to a smaller antenna size, given the slower reduction of Q_A_. As shown in Fig. 2B, the area above the fluorescence rise curves was larger in HL-grown cells at all actinic light intensities used. The comparison of the slopes of linear regression fits across the measured rate points indicates a 40% difference in PSII absorption capacity between those 2 conditions (Fig. 2C). This demonstrates that the loss in LHCII induces a strong decrease in the amount of light absorbed by HL-adapted *C. ohadii*. Nonetheless, this is a more limited effect than expected from the massive loss of LHCII observed by the sucrose density gradient assay. These seemingly conflicting observations may be resolved if the CBR protein associated with a large number of carotenoids would act as a PSII antenna that substitutes in part for the loss in LHCII., Alternatively, the LL-grown cells may exhibit already small supercomplex size or accommodate a substantial fraction of quenched, free LHCII which do not transfer their excitation energy to the PSII reaction centers.

To understand the effect of a lower PSII antenna size on the PSII electron transfer rate (ETR II) under continuous illumination, the PSII yield (Φ_PSII_) was measured after a 30-s illumination at various light intensities. The yield was then multiplied by the maximal PSII rate at these intensities to provide the ETR II. The results are shown in Fig. 2D and demonstrate that in LL-grown cells the saturation of PSII ETR is reached at about 400 μmol photons m^−2^ s^−1^, similar to other algae and plants. However, at HL-grown cells that lack the PSII antenna, the saturation is reached at much higher light intensities, well above 1,500 μmol photons m^−2^ s^−1^, and the ETR amplitude is higher than in LL cells.

### Absence of the q_E_ component of NPQ in *C. ohadii*

**Figure 3.**
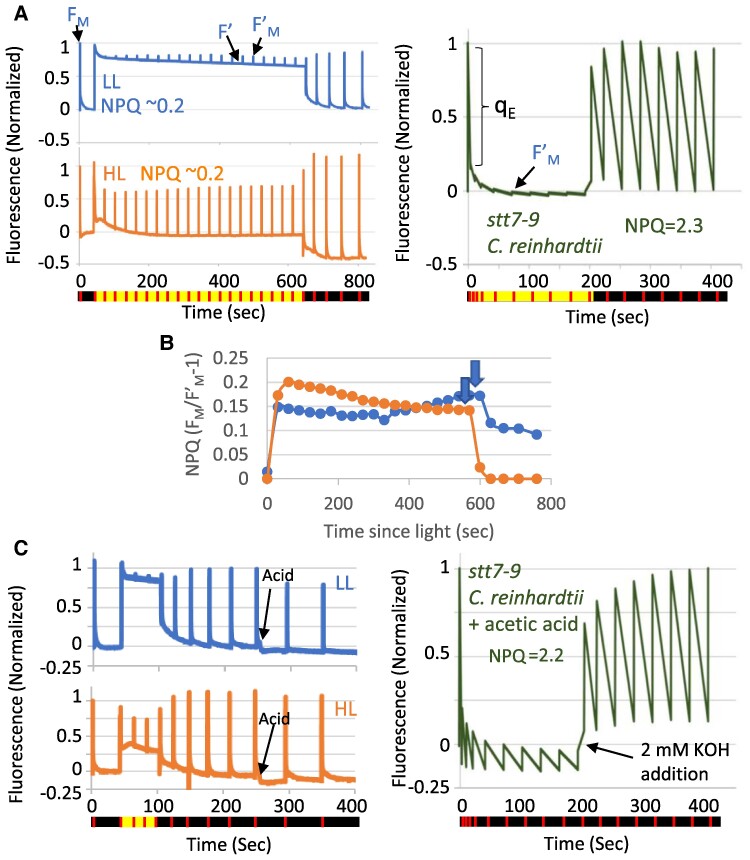
*Chlorella ohadii* exhibits virtually no protective NPQ. **A)** Left: Fluorescence traces of dark-adapted LL*-*grown (top) and HL*-*grown (bottom) *C. ohadii* cells, measured under 2,000 μmol photons m^−2^s^−1^. Black and yellow colors at the line below the figure indicate dark and actinic light conditions, respectively. The red line indicates the time points when saturating pulses were employed to induce maximum fluorescence (*F*_M_ and *F*′_M_). Low NPQ was detected as indicated by the little change in *F*′_M_ in response to exposure to high light. Right: Fluorescence traces of dark-adapted high-light-grown *stt7-9 C. reinhardtii* cells, exposed to 2,000 µmol photons m^−2^ s^−1^. Note the high NPQ levels. **B)** NPQ values were calculated from the fluorescence traces of dark-adapted LL and HL cells as shown in **(A)**. Down-pointing arrows indicate time of turning off of the actinic light. **C)** NPQ is triggered by high light and acidification of the lumen in *C. reinhardtii* but not in *C. ohadii.* Left: Fluorescence traces of dark-adapted LL-grown (top) and HL-grown (bottom) *C. ohadii* cells, measured under 2,000 μmol photons m^−2^s^−1^ or after the acidification of the media to pH 5.5. Black arrows indicate the point of acid addition. Right: Fluorescence traces of dark-adapted *stt7-9 C. reinhardtii* cells, exposed to acidic conditions. KOH was added to the cell following 200 s with no actinic light. The acidification of the medium induced high NPQ levels, mimicking the high-light activation of NPQ. This is reversed upon adding KOH, mimicking the light-off action (compare the left panels of **A** and **C**). Fluorescence was normalized between to *F*_M_ in the dark-adapted cells for all samples. All experiments are representative of at least 3 biological repeats.

Since the PSII peripheral antenna in green algae and plants is a major site of reversible NPQ, we investigated this photoprotective mechanism in *C. ohadii*. LL- and HL-grown cells were dark-adapted and then subjected to high actinic light intensity (2,000 µmol photons m^−2^ s^−1^), after which their fluorescence behaviors were recorded. Strikingly, both LL- and HL-grown cells showed a negligible NPQ response, as indicated by the limited decrease at *F*_M_′, with little if any NPQ calculated following the equation: NPQ = (*F*_M_/*F*_M_′)−1, where *F*_M_ and *F*_M_′ are the maximal fluorescence of dark-adapted cells and cells exposed to light, respectively, during a saturating light pulse (Fig. 3A). The decrease in *F*_M_′ was very limited in *C. ohadii* compared with (HL-grown) *C. reinhardtii* tested under similar light conditions (Fig. 3A). At 2,000 µmol photons m^−2^ s^−1^, NPQ reached a value of ∼0.2 (Fig. 3B), in *C. ohadii* compared with ∼2.3 in *Chlamydomonas* (*C. reinhardtii*), owing to its well-established q_E_ NPQ response of high amplitude, facilitated by the proton-sensing capabilities of LhcSR (Fig. 3A). When *C. ohadii* was exposed to even higher light intensities (3,000 µmol photons m^−2^ s^−1^) or when grown under strict photoautotrophic conditions, LL-grown cells developed only a limited NPQ response (∼0.7), with similar levels of NPQ in HL-grown cells (∼0.2) (Supplementary Figs. S1 and S2). Thus, under all tested conditions, virtually no light-induced q_E_ quenching was detected in *C. ohadii*.

q_E_ is the major and most rapid component of NPQ which responds to light in seconds and is activated by a pH decrease of the thylakoid lumen. Lumen acidification occurs due to the translocation of protons resulting from high photosynthetic electron flow under HL conditions. These protons bind conserved luminal glutamate residues in LhcSR (algae) and PsbS (plants) and activate q_E_. Even though *C. ohadii* lacks the gene encoding for the LhcSR protein and shows no evidence for PsbS expression (Treves et al. 2016; Levin et al. 2021, 2023), CBRs—which largely accumulate in HL-grown cells—harbor a conserved glutamate that may function as the proton sensor activating q_E_ (Levin et al. 2023; Levin and Schuster 2023). To exclude that there was a limitation to the lumen acidification due to the strong decrease of PSII antenna size and to assess the ability of CBR to trigger qE at low lumenal pH, we used the externally added acid method, as described in Tian et al. (2019). The fluorescence traces of LL- and HL-grown cells were followed after artificial lumen acidification to pH 5.5. In agreement with the above in vivo analysis of light-induced NPQ, lumen acidification had little effect on the fluorescence signal, indicating the absence of q_E_ activation in both LL- and HL-grown *C. ohadii* cells cultured in either mixotrophic (Fig. 3C) or strictly photoautotrophic (Supplementary Fig. S3) conditions. When the same assay was applied to *C. reinhardtii* cells as a positive control, fluorescence changes were observed, indicative of a strong q_E_ (Fig. 3C). The lack of q_E_ in *C. ohadii* agrees with the absence of the *lhcSR* gene and the lack of PsbS accumulation and mirrors the behavior of a *C. reinhardtii* mutant lacking the major NPQ effector, LhcSR3 (Tian et al. 2019), and the *psbS* mutant of Arabidopsis (Wilson et al. 2024). Importantly, we conclude that, despite a conserved glutamate (E131 in *C. ohadii*, E122 in *A. thaliana*) residue on the luminal side of the predicted CBR structure (Levin et al. 2023; Levin and Schuster 2023), this protein does not act as a pH-induced q_E_ trigger.

We have previously shown that besides the lack of q_E_ shown here, an additional NPQ contributor, the phosphorylation-induced state transitions or q_T_, is also absent in HL-grown *C. ohadii* cells (Levin et al. 2023). Together, these results indicate that *C. ohadii* does not rely on NPQ mechanisms to survive when grown under intense HL. While LL-grown cells still showed a very limited, slowly developing, NPQ effect, possibly due to q_I_ quenching, HL-grown cells were fully lacking in the 2 types of NPQ.

### HL-grown cells accumulate less damaging ROS than LL-grown cells under high red light illumination

**Figure 4.**
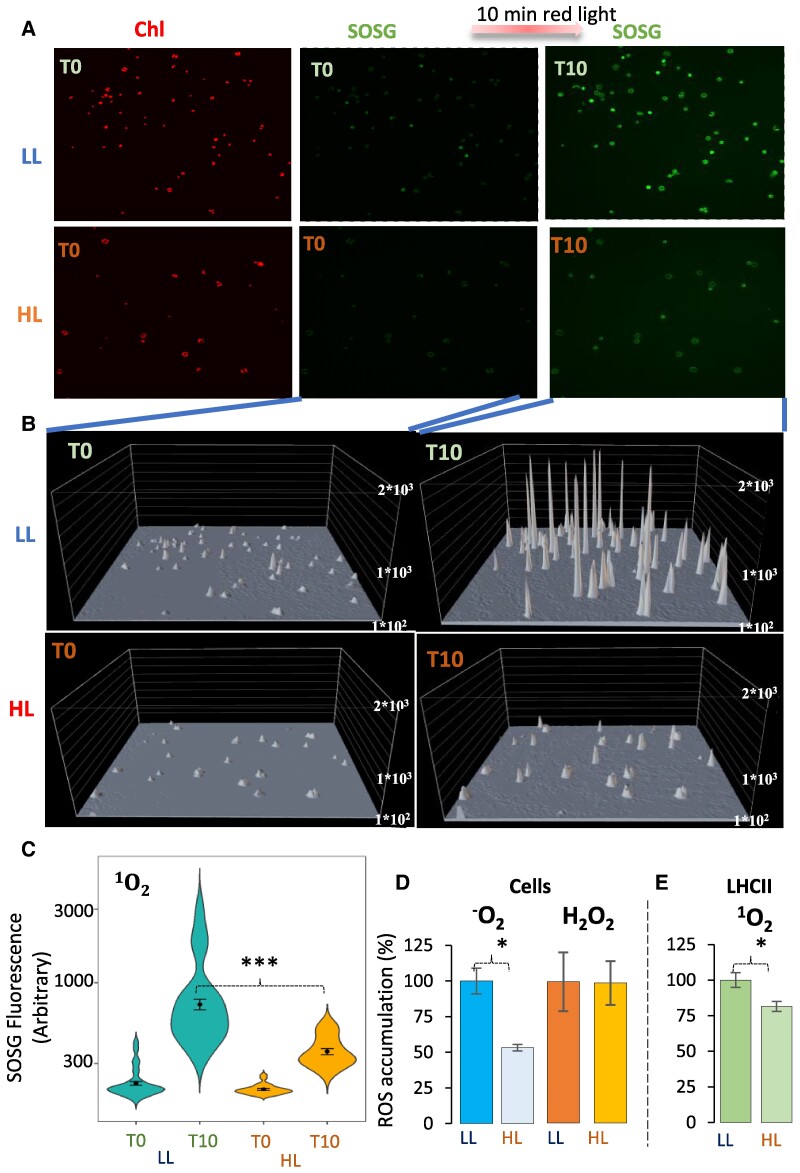
Decreased ^1^O_2_ accumulation in HL-grown *C. ohadii* upon high red light treatment. **A)** Confocal microscopy wide-field vision of low- and high-light (LL and HL)-grown cells. Their intrinsic chlorophyll fluorescence shown on the left panels was used to localize the chloroplasts. Note the decreased fluorescence of the HL-grown cells. In the middle and right panels, the fluorescence of the ^1^O_2_ marker SOSG is shown, originating from the same chloroplasts that are shown in the left panels. The SOSG fluorescence has been detected before (T0) and after 10 min (T10) of high red light treatment. **B)** Quantification of ^1^O_2_ SOSG signal of the chloroplasts shown in **(A)**. Only the SOSG fluorescence that overlaps the intrinsic chlorophyll fluorescent marking the chloroplast has been counted for the quantification (see Supplementary Fig. S4). **C)** Violin plots of ^1^O_2_ formation following 10 min of illumination with high-intensity red light in 54 and 24 individual chloroplasts of LL- and HL-grown cells, respectively. Post hoc comparisons between treatments show significant differences at T10. ***Bonferroni-adjusted *P* value < 0.000000005. The internal point indicates the mean value, and the error bars indicate the SE. The mean numbers are: LL T0: 223, LL T10: 723, HL T0: 204, HL T10: 358. Note the log scale of the *y*-axis. **D)** Accumulation of O^−^_2_ and H_2_O_2_ was quantified in LL- and HL-grown cells after 10 min of strong light illumination with NBT and 3,3′-DAB, respectively. The resulting precipitates were quantified based on color intensity with ImageJ. The data for HL cells are presented as a percentage of the formation in LL cells. Error bars represent the SD from 2 biological repeats. *Two-tailed Student’s *t* test *P* value < 0.05. **E)**^1^O_2_ accumulation in isolated LHCII complexes of LL- and HL-grown cells after 10 min of strong light treatment was measured by quantifying SOSG fluorescence. *Two-tailed Student’s *t* test *P* value < 0.05. Error bars represent the SD from 3 biological repeats.

**Figure 5.**
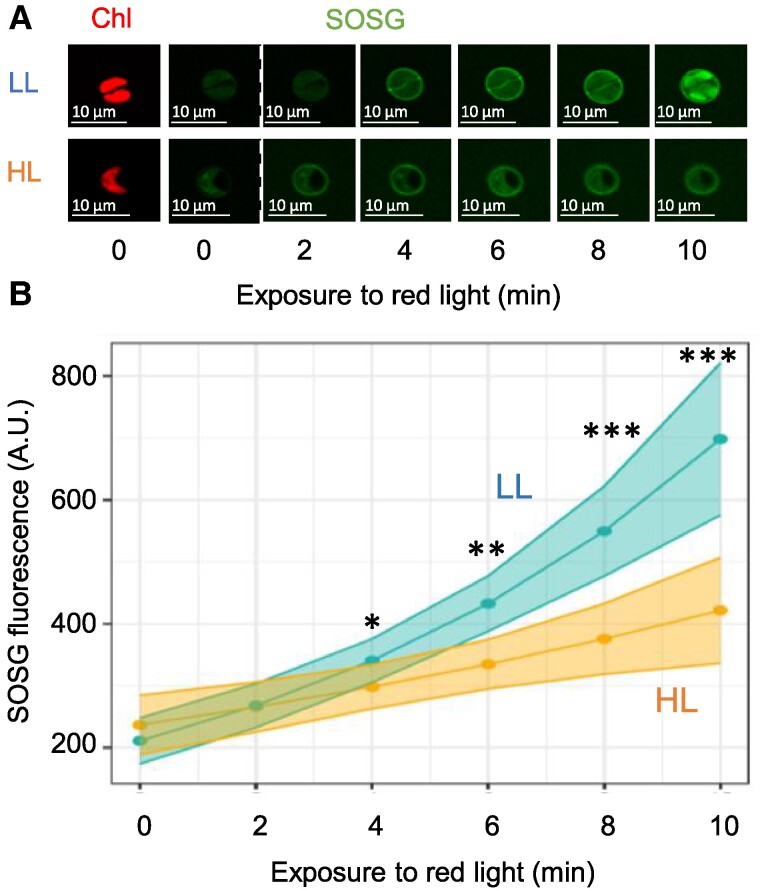
Kinetics of ^1^O_2_ production in *C. ohadii*. **A)** LL- and HL-grown cells were placed in a medium containing the ^1^O_2_ marker SOSG in the confocal microscope and chlorophyll fluorescence was detected to locate the chloroplasts in the cells (chl). The cells were then illuminated with strong red light for the times indicated at the bottom and the accumulated ^1^O_2_ was detected by the fluorescence intensity of SOSG. **B)** Quantification of the SOSG accumulation in 54 and 24 individual chloroplasts from LL- and HL-grown cells, respectively, as shown in **(A)**. Statistical analysis was performed with a linear mixed model of the log10-transformed response with light as fixed effect and time as both fixed and random effects. Shadowed area represents ±2 × SE. Mixed-model ANOVA showed a significant effect of time (*P* = 0.0027) and a significant interaction between light and time (*P* = 3.215E−06). Post hoc comparisons between treatments in each time point resulted in significant differences in time points 4, 6, 8, and 10. Bonferroni-adjusted *P value < 0.05, **P value < 0.0000005, ***P value < 0.000000005.

To quantify the photoprotective capacity of *C. ohadii* to limit ROS accumulation in the absence of NPQ, we used confocal microscopy coupled with the detection of Singlet Oxygen Sensor Green (SOSG). This dye emits green fluorescence upon interaction with ^1^O_2_ (Supplementary Fig. S4). LL- and HL-grown cells were incubated with SOSG and exposed to 10 min of high red actinic light to facilitate photosynthetically induced ^1^O_2_ formation, followed by quantification of the accumulated ^1^O_2_. Illumination with red light (∼600 nm) was chosen to prevent SOSG autofluorescence (excitation at 488 nm) (Prasad et al. 2018). HL-grown cells accumulated significantly lower levels of ^1^O_2_ than LL-grown cells (Fig. 4, A–C). To evaluate the contribution of ^1^O_2_ that was generated via photosynthesis-related processes to this difference, we analyzed the SOSG fluorescence that was emitted specifically within the chloroplast (Supplementary Fig. S4). The analysis of 24 and 54 individual chloroplasts from LL- and HL-grown cells, respectively, showed that individual chloroplasts of LL-grown cells accumulated much higher levels of ^1^O_2_ (Fig. 4C), with a 324% increase following 10 min of red light illumination, while in HL-grown cells, the increase was limited to an average of 175% (Fig. 4C). These results are in line with the increased accumulation of O_2_^•−^ in LL-grown when compared with HL-grown algae (Fig. 4D). In contrast, cells from the 2 light regimes accumulated similar amounts of H_2_O_2_ after 10-min illumination with strong white light, as measured with nitro blue tetrazolium (NBT) and 3,3′-diaminobenzidine (DAB), which precipitate upon interaction with O_2_^•−^ and H_2_O_2_, respectively (Fig. 4D) (Levin et al. 2023). Kinetic analyses further confirmed that LL-grown cells accumulated ^1^O_2_ at a significantly higher rate (Fig. 5). Moreover, a substantial proportion of chloroplasts of LL-grown cells accumulated 10 times more ^1^O_2_ than those of HL-grown cells (Fig. 4B).

To determine how changes in peripheral antenna between the 2 light growth conditions were involved in these differences in photosensitivity and ROS production, ^1^O_2_ production was compared between detached LHCII fractions (Fig. 2A) of LL- vs. HL-grown cells at the same chlorophyll concentration. Importantly, detached LHCII fractions from HL-grown cells also contain large amounts of carotenoids and CBR, which may contribute to reduced ^1^O_2_ accumulation via direct ^1^O_2_ scavenging (Levin et al. 2023). The results shown in Fig. 4E demonstrate that the CBR-containing detached LHCII fractions from HL-grown cells indeed accumulated less ^1^O_2_ compared with the same fraction from LL-grown cells. However, the difference was smaller than in whole-cell measurements, suggesting that both the antenna size difference and the differences in the detached antenna properties or in accumulation of CBR and carotenoids had a paramount influence on the rate of ROS synthesis upon photosynthesis.

### Cyclic electron flow is highly activated in HL-grown cells

**Figure 6.**
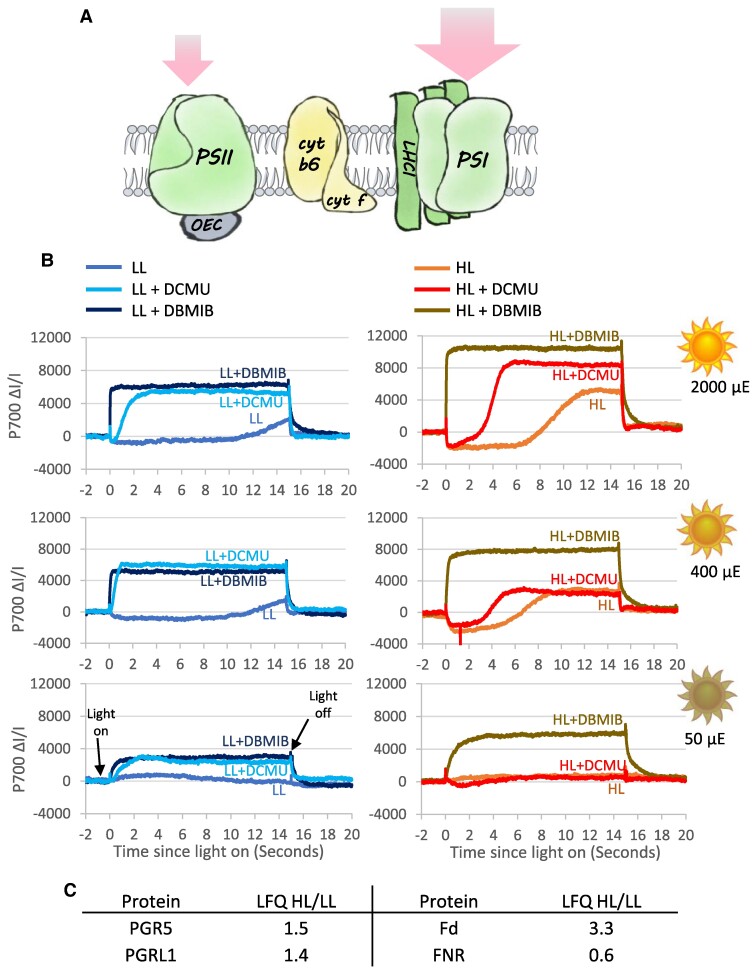
P_700_ redox measurements suggest a strong increase of CEF efficiency upon high-light acclimation of *C. ohadii*. **A)** In HL-grown *C. ohadii*, the LHCII is removed from PSII, while PSI maintains all or most of its antenna subunits. As a result, PSI is expected to absorb higher quantities of light energy. **B)** P700 oxidation kinetics analysis in dark-adapted cells shows that in the presence of DCMU which blocks LEF, PSI of HL-grown cells is oxidized at a slower rate compared with that of LL-grown cells in all tested light intensities. When CEF is blocked by DBMIB, similar PSI oxidation rates are observed in both growth conditions. Note that in LL-grown cells, CEF becomes apparent only by HL treatment, while in HL-grown cells, CEF is highly activated at all light intensities, and LEF is not activated at very low light intensities. 6 mm methyl viologen was added to all samples. The graphs are representative of at least 3 biological repeats. **C)** PGR5, PGRL1, and Fd accumulate in HL-grown cells, while FNR accumulates in LL-grown cells, as measured with LFQ –LC–MS/MS.

A decrease in PSII antenna relative to that of PSI creates a favorable environment for CEF around PSI (Fig. 6A) (Tagawa et al. 1963; Joliot and Joliot 2005; Alric 2015; Nawrocki et al. 2019b). This led us to assess the kinetics of light-induced P_700_ oxidation to determine whether CEF exhibited a different behavior in LL- vs. HL-grown *C. ohadii*. We used the P_700_ cation absorption at 830 nm as a proxy for the activity of CEF, measured when PSII is inactivated by the addition of the herbicide DCMU, compared with the 830 nm absorption change when both CEF and LEF are blocked by DBMIB—a cytochrome *b*_6_*f* inhibitor (Nawrocki et al. 2019a). Drastically different behaviors of P_700_ oxidation were observed between the LL- and HL-grown algae (Fig. 6B). When exposed to the highest tested actinic light intensities in the presence of DCMU, a long lag in P_700_ oxidation was noted when cells were grown in HL, suggesting a high CEF activity (Fig. 6B upper panels). Remarkably, this lag was still observed in the presence of methyl viologen, an electron acceptor from PSI, which also decreases the frequency of back reactions while preserving CEF to some extent (Nawrocki et al. 2019a; Joliot et al. 2022). The half-time of PSI oxidation by CEF under these conditions of extremely strong illumination, as determined by the signal obtained following the addition of DCMU, reached 4 s, at least 4 times longer than in LL-grown *C. ohadii*, and more than one order of magnitude longer than in *Chlamydomonas* (Nawrocki et al. 2019a). Indeed, the steady state of P_700_ oxidation under continuous illumination at 2,000 µmol photons m^−2^ s^−1^—itself a commonly used proxy for CEF activity (Takahashi et al. 2013; Alric 2014)—was only around 80%, indicating that even in the absence of PSII activity, a very high rate of CEF can be maintained in *C. ohadii* adapted to HL conditions (Fig. 6B). As expected, this effect was abolished when DBMIB rather than DCMU was added, demonstrating the requirement of electron flow through the cytochrome *b*_6_*f* to maintain CEF. Finally, at lower actinic light intensities in the absence of inhibitors, there was no accumulation of P_700_^+^ due to its efficient reduction by PSII (Fig. 6B). The effect of DCMU was visible in cells grown in LL, while no effect was detected in HL-grown *C. ohadii*, further indicating that a highly efficient CEF maintains P_700_ mostly reduced in the latter condition. CEF is highly active in HL-grown cells despite the accumulation of Flvs which oxidize the acceptor side of PSI and thus should counter the PSI reduction by CEF and increase PSI oxidation rates, suggesting that CEF is a major alternative electron flow route in HL-grown *C. ohadii*. The inability to completely oxidize P_700_ at LL in the presence of DBMIB hints to additional pathways of P_700_^+^ reduction, as substantiated by the relatively rapid reduction of the cation at the end of the illumination period.

The stimulation of CEF in HL-grown cells was consistent with MS data. According to our proteomics analysis, proton gradient regulation 5 (PGR5) and PRG5-like photosynthetic phenotype 1 (PGRL1), which regulate CEF (Nawrocki et al. 2019b), showed enhanced accumulation in HL-grown cells (1.5-fold for both enzymes) (Fig. 6C). Also, the enhanced accumulation of ferredoxin (Fd) (3.7-fold), together with the decrease in Fd-NADP-reductase (FNR) levels (0.4-fold change), suggested a shift toward CEF rather than toward LEF (Fig. 6C).

Together, these results imply that CEF is induced in HL-grown cells and is a major electron pathway during both LL and HL exposure Therefore, aside from the natural requirement to balance the photosynthetic electron flow according to the imbalanced absorption cross-sections of PSII and PSI, the highly active CEF in HL-grown cells likely contributes an extra ATP generation that would be required in these environmental conditions.

## Discussion

### An efficient NPQ-independent photoprotective mechanism underlines *C. ohadii* remarkable resistance to high-light stress

**Figure 7.**
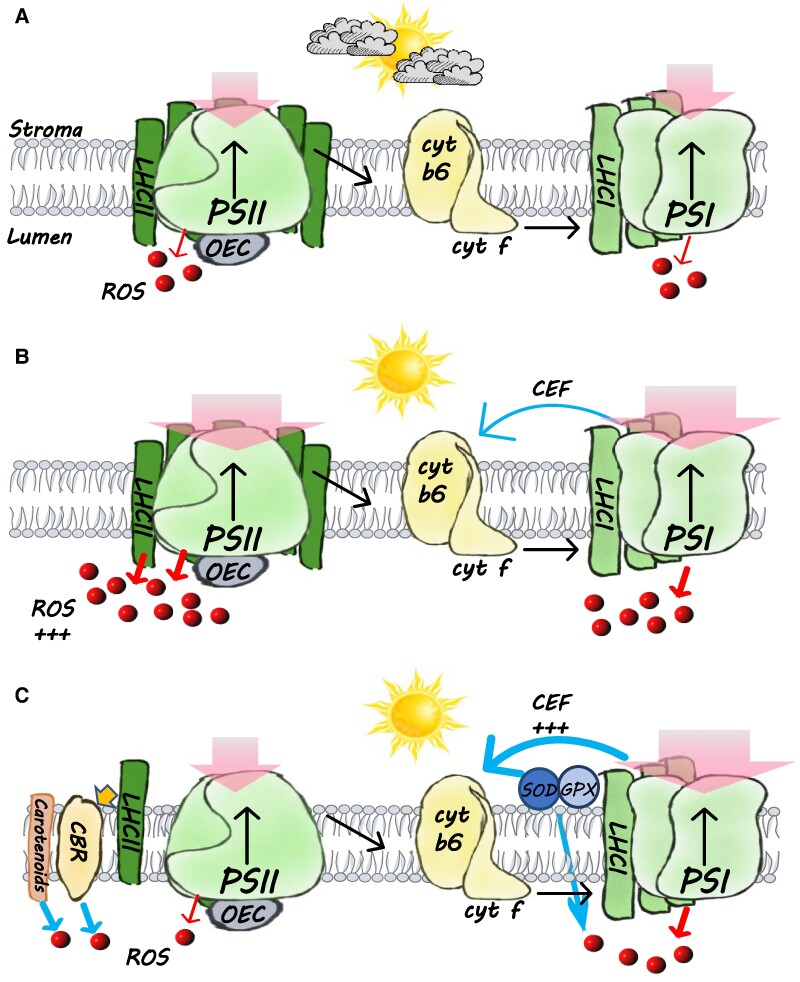
*Chlorella ohadii* does not require NPQ for photoprotection. **A)** Under LL conditions, LEF is favored and small amounts of ROS are generated. **B)** Upon a shift to HL conditions, large amounts of ROS are produced in the PSII and by its antenna the LHCII. CEF around PSI is relatively low. **C)** To avoid light-induced oxidative damage, HL-grown *C. ohadii* eliminates the LHCII, massively reducing the absorption of excess light energy. Additionally, carotenoids, SOD, GPX, and possibly the CBR protein and other enzymatic antioxidants act as ROS scavengers. CBR may quench light energy that is absorbed by the detached LHCII, before their degradation. CEF is highly active—at least in the absence of LEF—and may provide extra ATP for metabolic processes.

Extremophile species are fascinating forms of living matter, even more so that—their growth environments being exacerbated versions of stressful situations—they may serve as models for the discovery of engineering strategies for the generation of more resistant crops. Recently, we highlighted the importance of including high-light-tolerant organisms in future photoprotection research (Levin and Schuster 2024), considering the possibility they harbor highly specialized protective mechanisms, as demonstrated in this work. Photoprotection is thought to be a primary photosynthesis defense mechanism. The various forms of NPQ-controlled photoprotection processes are widely studied, from their molecular mechanism, by numerous molecular and chemical effectors, to their impact on gene expression (for recent reviews, see Niyogi and Truong (2013), Murchie and Ruban (2020), and Bassi and Dall’Osto (2021)). While similar NPQ pathways are widespread in most plants and algae, the current work shows that an extremely light-tolerant microalga, *C. ohadii*, does not require any NPQ to thrive under constant extreme HL intensities (Fig. 7). When these cells are grown at LL intensities, PSII harvests light both through its core and peripheral antennae and generates ROS, as the membranes contain limited amount of carotenoids (Fig. 7A). However, when these LL-grown cells are exposed to excess light intensities before being acclimated to HL conditions, even more ROS are produced, leading to protein oxidation and a high-light acclimation response (Fig. 7B). Indeed, this acclimation is efficient: we observed large differences in the levels of oxidized peptides between the LL- and HL-grown cells after an acclimation period of 24 h, suggestive of higher levels of protein damage in LL-grown cells (Fig. 1C). Acclimation to HL conditions leads to a major loss in PSII peripheral antennae, while several antioxidant enzymes, CBR, and a large number of carotenoids accumulate (Levin et al. 2021, 2023). The combined effect of these processes is a marked decrease in the generation and accumulation of ROS, which results in less protein oxidation (Fig. 7C). Thus, although the elimination of the LHCII in HL-grown *C. ohadii* leads to a potential loss of NPQ sites, it majorly reduces the absorption cross-section of PSII which, in turn, keeps ROS production to a low level under high light. Together with ROS detoxification carried out by the increased accumulation of enzymatic and nonenzymatic antioxidants, it drastically reduces the rate of damage that could be caused by the concurrent energy conversion process (Fig. 7).

### Minimizing light absorption may be a more efficient approach to photoprotection than NPQ induction under steady illumination

A drop in LHCII is potentially a risky acclimation strategy for photoprotection upon exposure to high-light conditions for at least 2 reasons. It leads to a loss in most NPQ regulatory processes which are critical to mitigate the effects of abrupt changes in light intensities under a fluctuating light regime. In addition, it decreases the light-harvesting capabilities during LL periods. However, *C. ohadii* thrives in the soil crusts of the desert under direct sunlight, where few clouds or vegetation can provide shading. Therefore, it is placed in a very stable light environment with extended periods under constant HL. In these conditions, the small core antenna of PSII provides enough light energy to fuel PSII-mediated electron transfer and the elimination of LHCII allows a drastic reduction in light-induced ROS production (Fig. 7). It is of note that alongside LHCII elimination, the loss of NPQ in *C. ohadii* also is driven by the absence of the *lhcSR* gene, the PsbS protein, and the state transition-dependent q_T_ (Treves et al. 2016; Levin et al. 2023). Thus, *C. ohadii* provides a remarkable demonstration of what NPQ may not be suited for: it may not deliver an appropriate photoprotection against long-lived illumination at very high light intensities, as encountered among extremophiles. In support of this notion, relatively low NPQ was measured in other photosynthetic organisms that are native to the desert (Eppel et al. 2013, 2014; Wu et al. 2018). Nevertheless, more data are needed to determine whether the lesser role of NPQ is common among different species. In this view, NPQ should be regarded as a series of photoprotection responses that evolved with oxygenic photosynthesis exposed to fluctuating light ranging from frequent nonsaturating illumination to transient exposures to saturating light as is the case for most crop species and microalgae studied so far. This interpretation is in line with the recent data of photosynthetic response in the frequency domain, with the strongest NPQ effect taking place on a ∼minutes timescale (Niu et al. 2023). Following this interpretation, it would be interesting to determine whether the limited NPQ response in *C. ohadii* leads to increased sensitivity to fluctuating light conditions.

The extent of the antenna loss, estimated from MS data upon solubilization of photosynthetic membranes, looked more extensive than the substantial but more limited ∼40% functional decrease in PSII light-harvesting capacity during a switch between LL and HL growth (Figs. 1 and 2). This apparent discrepancy may stem from a functional antenna size in LL conditions being smaller than suggested by the amount of LHCII in the sample, in which case a fraction of this peripheral antenna would be disconnected from the reaction centers but aggregated in a quenched state. These quenched LHCII would not contribute to PSII cross-section, and they would barely affect the *F*_V_/*F*_M_ in cells, and their loss upon HL acclimation would be consistent with the decrease of chlorophyll content. Notably, a *truncated light-harvesting antenna2 C. reinhardtii* mutant which is deficient in LHCs displayed a similar phenotype to *C. ohadii* HL-grown cells in terms of reduced chlorophyll content and higher chlorophyll *a/b* ratio, a reduction of ∼35% in the functional antenna size, and higher photosynthetic activity under strong illumination (Kirst et al. 2012), suggesting that elimination of LHC may be beneficial in other species too when exposed to a high-light regime. As an alternative hypothesis, a substitution mechanism for LHCII may take place under HL conditions: another LHC would contribute to the PSII absorption cross-section, CBRs being the primary candidates given their increased abundance. In the future, time- and spectrally resolved fluorescence methods could reveal the lifetimes of chlorophyll excited states in each fraction of the antenna in vivo and help decipher which of the 2 hypotheses is correct.

### Minimizing light absorbance while accumulating antioxidants leads to reduced oxidative damage

In addition to lowering ROS production, the decrease in LHCII content in *C. ohadii* is accompanied by the accumulation of antioxidants such as carotenoids (Levin et al. 2021, 2023) and specialized enzymes, specifically GPX, SOD, and perhaps CBR (Fig. 1A). The role of the latter protein remains elusive, but the marked increase in accumulation under HL suggests a key role in *C. ohadii* photoacclimation (Fig. 1A). Despite its structural similarities with PsbS and LhcSR, CBR does not induce q_E_ or other NPQ processes via lumen acidity sensing (Fig. 3B and Supplementary Fig. S3). It was suggested that CBR binds carotenoids (Levy et al. 1993) and, indeed, it was found to co-localize with accumulated carotenoids and the detached LHCII fraction of HL-grown *C. ohadii* (Levin et al. 2023). Carotenoids are efficient ^1^O_2_ scavengers (Triantaphylidès and Havaux 2009), and CBR may contribute to these activities, while it is bound to carotenoids. Moreover, the large accumulation of carotenoids in the thylakoid membrane of HL-grown *C. ohadii*, predominantly zeaxanthin and lutein, may contribute to this effect (Levin et al. 2023). In addition to being a ^1^O_2_ target, CBR may be an internal UV light screen, expressed by the cells to prevent donor-side damage to PSII.

The overall level of detectable ROS was significantly decreased upon illumination of HL-grown alga. Given the elimination of the PSII antenna and antioxidant accumulation, it is likely that the decline in ROS levels is a product of the smaller PSII absorption cross-section and of the detoxifying effect of the upregulated O_2_^•−^-scavenging enzymes and the ^1^O_2_-scavenging carotenoids (Fig. 7). More specifically, the smaller cross-section results in a mild decrease in the amount of absorbed light, which limits ROS synthesis (Santabarbara et al. 1999, 2001). Then, the decreased concentration of nascent ROS due to scavenging activity or using CBR-bound carotenoids as an oxidation target minimizes the overall accumulation of these deleterious species. Additionally, rapid turnover of damaged proteins in HL-grown cells may also contribute to the reduced accumulation of oxidized peptides.

### What is the role of highly active CEF in *C. ohadii*?

Finally, CEF around PSI is often thought of as a transient process that occurs before the Calvin–Benson cycle becomes active at the onset of illumination of vascular plants. In addition, because CEF drives acidification of the thylakoid lumen, it contributes to photoprotection of PSII through NPQ activation but also to protecting PSI from photoinhibition since it slows down P_700_^+^ re-reduction by the cytochrome *b*_6_*f* complex (Harris and Boekema 2018). Last, it has also been implicated at a means to maintain ATP production in anaerobic conditions in microalgae, when mitochondrial respiration is inhibited (for recent reviews on CEF, see Suorsa (2015), Labs et al. (2016), and Nawrocki et al. (2019b)). The finding that CEF is highly active particularly in HL-grown *C. ohadii* calls for a specific role in this alga. Since little or no lumen acidification-induced NPQ was detected in the alga (Fig. 3B), it shows that this highly active CEF is not used to boost PSII photoprotection by the classic NPQ mechanism. Additionally, P_700_ is primarily maintained in its neutral form even under high light (Fig. 6B) (Caspy et al. 2021). These findings suggest that CEF is not employed to boost lumen acidification—neither for PSII, nor for PSI photoprotection.

It remains that CEF in *C. ohadii* should enhance ATP synthesis in HL conditions. This may be needed to cope with high rates of photoinhibition under desert growth conditions and the heavy energetic cost of PSII repair. Generally speaking, CEF-generated ATP would provide the required energy for keeping with intracellular metabolism in nongrowing or slowly growing conditions, whether for protein and DNA repair, protein translation, and energy storage for dark periods.

It is a long-standing technical difficulty in the field of functional photosynthesis to ascertain that transient CEF in the absence of PSII activity—as is the case in our measurements—reflects the situation when LEF is functioning (Fan et al. 2016). The long-lived CEF at the onset of illumination in *C. ohadii* could nonetheless serve as a model for the development of methodologies for its quantification, a critical feat for photosynthesis physiology in the coming years.

## Materials and methods

### Cell culture and acclimation to low- or high-light conditions

Exponential-phase *C. ohadii* cells were diluted to optical density (OD)_750nm_ 0.2 in a final volume of 400 mL TAP medium and then split: 200 mL was placed under LL (50 µmol photons m^−2^ s^−1^) conditions and 200 mL was placed under HL (2,000 µmol photons m^−2^ s^−1^) conditions, in 1 L Erlenmeyer under shaking (100 rpm), for 24 h, at 28 °C. To prevent self-shading, the cells were maintained below an OD_750nm_ value of 0.8 by diluting them to OD_750nm_ 0.2 every 8 h during the growth period. After 24 h, the LL- and HL-grown cells were harvested for further experimentation. For experiments under strict photoautotrophic conditions (Supplementary Figs. S1 and S3), the cells were grown in a Tris-phosphate medium, with no acetate as a carbon source.

*Chlamydomonas reinhardtii* stt7–9 strain, lacking the kinase activity required for state transitions, was grown in continuous 400 µmol photons m^−2^ s^−1^ condition in MIN media (Hui et al. 2023) under strict photoautotrophic conditions.

### Photosynthetic complex separation by sucrose density gradient centrifugation

LL- and HL-grown cells were pelleted and washed twice with sucrose (300 mm)–Tris-tricine (15 mm each pH 8)–NaCl_2_ (15 mm) (STN) buffer. The cells were broken in a MicroFluidizer (50 psi); unbroken cells were pelleted (15,000 g, 10 min, 4 °C). The supernatant was collected, and thylakoid membranes were pelleted in an ultracentrifuge (250,000 g, 2 h, 4 °C). Thylakoid membranes were resuspended in 25 mm MES (pH 6.5) to a chlorophyll concentration of 0.2 or 0.4 mg/mL for LL- or HL-grown samples, respectively, and solubilized with 1% *n*-dodecyl-α-D-maltopyranoside (α-DDM) for 15 min, on ice, under dark conditions. Insolubilized material was pelleted (9,500 g, 10 min, 4 °C), and the supernatant was loaded on a sucrose density gradient (20 mm MES pH 6.5, 0.02% α-DDM, 4% to 45% sucrose). Individual complexes were separated by ultracentrifugation (100,000 g, 20 h, 4 °C).

### Functional analyses

LL- and HL-grown cells were pelleted and resuspended in fresh medium to the equivalent of 10 μg/mL chlorophyll for PSII chlorophyll fluorescence analysis, or 50 μg/mL for P_700_ (PSI) absorption analysis. The cells were dark-adapted for at least 30 min with air bubbling, then loaded to a quartz cuvette, and analyzed with a Dual-PAM-100 fluorometer (Walz, Germany). Red actinic light was used in all measurements.

#### PSII electron transport rate saturation curve

After exposure of LL- and HL-grown cells to different light intensities for 30 s, the PSII operating efficiency (Φ_PSII_) was measured using the following equation: Φ_PSII_ = (*F*_M_′ − *F*′)/*F*_M_′. PSII ETR was calculated following the equation: ETR(II) = σ_II_ · Φ_PSII_, where σ_II_ is the maximal, light-limited rate of PSII at each light intensity (“PSII antenna size”). The latter is the reciprocal of the area above fluorescence–DCMU curve, delimited by *F*_M_ value normalized to 1 and by the *t*_0_.

#### NPQ analysis

The fluorescence traces of LL- and HL-grown cells were monitored upon exposure to various light intensities (50, 400, 2,000, and 3,000 µmol photons m^−2^ s^−1^) for 10 min, followed by 3 min of darkness for recovery. When indicated, acetic acid (1 mm final) was added directly to the cuvette to induce q_E_ (Tian et al. 2019). NPQ was calculated following the equation: NPQ = (*F*_M_/*F*_M_′)−1 in both *C. ohadii* and *C. reinhardtii*.

#### P_700_ redox measurements

P_700_ oxidation kinetics were monitored in LL- and HL-grown cells upon illumination with various light intensities: 50, 400, or 2,000 μmol photons m^−2^ s^−1^, in the presence or absence of the photosynthetic electron transfer inhibitors 3-(3,4-dichlorophenyl)-1,1-dimethylurea (100 μM, DCMU) or 2,5-dibromo-3-methyl-6-isopropylbenzoquinone (100 μM, DBMIB). Methyl viologen (6 mm) was added to all samples to prevent charge recombination in PSI.

### ROS accumulation assay

#### Live-cell ^1^O_2_ quantification with SOSG

LL- and HL-grown *C. ohadii* cells were pelleted and resuspended to OD_750nm_ 0.2 in TAP medium containing 50 μM SOSG. Subsequently, the cells were incubated for 30 min under LL conditions, at 28 °C, and pelleted and resuspended in fresh TAP medium. To visualize the formation of ^1^O_2_, the cells were loaded to a spinning disk confocal microscope (Nikon Eclipse Ti2, Confocal scanner unit Yokogawa CSU-W1, Camera Photometrics PrimS-BSI). Light stimulation was achieved using a red LED integrated into the microscope, providing an intensity of 3.9 mW (660 nm) for a duration of 1–10 min. The SOSG was excited at 488 nm, and the fluorescence emission was recorded at 530 nm. Chlorophyll fluorescence was recorded with excitation at 430 nm and emission at 680 nm to identify the chloroplasts. IMARIS software (Oxford Instruments) was employed to reconstruct the chloroplast structure based on the chlorophyll fluorescence. Subsequently, only SOSG fluorescence that corresponded to the reconstructed chloroplast was measured, ensuring a specific and accurate assessment of the effects of light stress on the observed photosynthetically derived ^1^O_2_ formation. The graphs were prepared with the R environment for statistical computing and graphics (https://www.R-project.org/) using the ggplot2 package (Wickham 2016). The linear mixed model was calculated with the lme4 package (Bates et al. 2015). SEs and post hoc analysis were calculated using the emmeans package (https://CRAN.R-project.org/package=emmeans). Mixed-model ANOVA was used to determine statistical significance (see Fig. 5B).

#### ^1^O_2_ accumulation in isolated LHCII complexes

Isolated LHCII from LL-grown cells and isolated LHCII together with CBR from HL-grown cells were resuspended in STNM buffer (50 mm Tricine–KOH pH 7.6, 15 mm NaCl_2_, 300 mm sucrose, and 5 mm MgCl_2_) with SOSG (10 mm) to a final chlorophyll concentration of 0.028 μg/μL. The samples were loaded onto a black 96-well plate, and SOSG fluorescence was measured before and after 10 min of light illumination in a fluorescence plate reader (ClarioStar 500, BMG Labtech, Ortenberg, Germany) with excitation at 488 nm and an emission range of 530 to 600 nm. The samples were placed on ice during the illumination period to avoid protein denaturation. STNM buffer with SOSG was used as a blank to measure and exclude the background SOSG fluorescence. Three biological repeats were tested for each treatment condition. Two-tailed Student’s *t* test was used to determine statistical significance (*P* value < 0.05).

#### O_2_^•−^ and H_2_O_2_ accumulation in LL- and HL-grown *C. ohadii* cells

NBT, which upon reduction creates blueish–greyish insoluble precipitates, and 3,3′-DAB, which upon oxidation by H_2_O_2_ becomes dark brown, were used to measure O_2_^•−^ and H_2_O_2_ accumulation in LL- and HL-grown *C. ohadii* cells. The cell OD_750nm_ was adjusted to 0.7. One milliliter of each sample was subjected to a 10-min incubation under HL conditions, with the addition of either 5 mm NBT or 5 mm DAB. Thereafter, chlorophyll was extracted with 100% methanol, at 40 °C. Cells were then pelleted (3000 g, 5 min) and resuspended in 200 μL methanol (100%). The resulting cell suspension was applied as spots onto the Whatman 3 paper filter. ROS were quantified with ImageJ, based on the color intensity. Two-tailed Student’s *t* test was used to determine statistical significance (*P* value < 0.05).

### MS whole-cell sample preparation and proteolysis

Three biological repeats of LL- and HL-grown *C. ohadii* cells were harvested, washed twice with STN, and broken under 50 psi with a MicroFluidizer. Samples were suspended in 8.5 m urea, 100 mm ammonium bicarbonate, and 10 mm DTT. Protein content was estimated using Bradford readings. The samples were reduced (60 °C, 30 min), modified with 35.2 mm iodoacetamide in 100 mm ammonium bicarbonate (room temperature for 30 min in the dark), and digested in 1.5 m urea and 17.6 mm ammonium bicarbonate with modified trypsin (Promega), overnight, at 37 °C, at a 1:50 (M/M) enzyme-to-substrate ratio. A second digestion with trypsin was performed for 4 h, at 37 °C, at a 1:100 (M/M) enzyme-to-substrate ratio. The tryptic peptides were desalted using Oasis HLB 96-well µElution Plate (Waters), dried, and resuspended in 0.1% formic acid.

### MS analysis

The resulting peptides were analyzed by LC–MS/MS using a Q Exactive HFX mass spectrometer (Thermo) fitted with an HPLC capillary (Ultimate 3000, Thermo Scientific). The peptides were loaded in solvent A (0.1% formic acid in water) on a homemade capillary column (30 cm, 75-micron ID) packed with Reprosil C18-Aqua (Dr. Maisch GmbH, Germany). The peptide mixture was resolved with a 5% to 28% linear gradient of solvent B (99.99% acetonitrile with 0.1% formic acid) for 180 min, followed by a gradient of 28% to 95% for 15 min, and then 15 min at 95% acetonitrile with 0.1% formic acid in water at flow rates of 0.15 μL/min. MS was performed in a positive mode (m/z 350–1,200, resolution 120,000 for MS1 and 15,000 for MS2), using repetitively full MS scan followed by high collision dissociation (at 27 normalized collision energy) of the 30 most dominant ions (>1 charges) selected from the first MS scan. The AGC settings were 3 × 10^6^ for the full MS and 1 × 10^5^ for the MS/MS scans. A dynamic exclusion list was enabled with an exclusion duration of 20 s.

### MS data analysis

Raw data were processed with MaxQuant version 2.5.2 (Tyanova et al. 2016) for protein accumulation analysis and MSfragger version 4.0 (Kong et al. 2017) via FragPipe version 21.1 (https://fragpipe.nesvilab.org/) for detection of tryptophan oxidized peptides. In the absence of a fully annotated proteome for *C. ohadii*, we utilized a combined dataset of protein sequences in our identification process. This dataset includes all available Chlorella protein sequences (Tax ID = 3701) downloaded from UniProt as of July 2019, comprising a total of 20,973 sequences. Additionally, we incorporated all available *C. ohadii* protein sequences (11,470) from the NCBI, as downloaded on October 2023. All searches included enzymatic cleavage with trypsin (cleavage after K/R but not P) and 2 missed cleavages and a clip of N-term methionine unless specified otherwise. Cysteine carbamidomethyl (+57.02146) was set as a fixed modification, and methionine oxidation (+15.994915) and protein N-terminal acetylation were set as variable modifications.

MaxQuant searches were performed using tryptic digestion mode with a minimal peptide length of 7 amino acids. Search criteria included up to 2 missed cleavages, precursor and fragments tolerance of 20 ppm, oxidation of methionine, and protein N-terminal acetylation set as variable modifications. Default settings were used for all other parameters. Candidates were filtered to obtain a false discovery rate (FDR) of 1% at the peptide and protein levels. For quantification, the match between runs (MBR) module of MaxQuant was used with the LFQ normalization method enabled.

Identification and quantification of tryptophan oxidation were performed using FragPipe’s “LFQ-MBR” workflow. The workflow default settings were used with the additions of tryptophan oxidation (+15.994915) and deoxidation (+31.989829) as variable modifications for identifying tryptophan oxidation.

Further analysis was performed using the Perseus software v.2.0.11 (Tyanova et al. 2016). LFQ intensity values were used to calculate the relative abundance of total proteins and tryptophan oxidized peptides in LL and HL whole-cell samples. The results were filtered to exclude contaminants, reverse, and only identified by site proteins. LFQ values were transformed (log2) and filtered to include only proteins that were detected in 3 repeats of at least 1 group: LL or HL samples. Missing values were then added from a normal distribution (https://cox-labs.github.io/coxdocs/replacemissingfromgaussian.html). A two-sample *t* test was used to determine the statistical significance of differences between groups, applying a permutation-based FDR of 0.05 and an S0 parameter of 0.1. iBAQ (Schwanhäusser et al. 2011) was used to determine the abundance of a given protein in HL-grown cells. A protein with a higher iBAQ value is more abundant compared with a protein with a low iBAQ value. The proteins were listed according to their average iBAQ values (high to low) in HL-grown cells. The percentile of the protein was determined by dividing its number (position) on the list, by the total number of proteins, and multiplying by 100. For example, if protein X is 40th on the list, and a total of 4,000 proteins were detected in the analysis, then protein X is in the 1st percentile, i.e. in the top 1% most abundant proteins in the cell.

## Supplementary Material

kiae608_Supplementary_Data

## Data Availability

The MS proteomics RAW data have been deposited to the ProteomeXchange Consortium via the PRIDE (Perez-Riverol et al. 2022) partner repository with the dataset identifier PXD053918.

## Supplementary data

The following materials are available in the online version of this article.

**Supplementary Figure S1.** *Chlorella ohadii* lacks NPQ under strict photoautotrophic conditions.

**Supplementary Figure S2.** *Chlorella ohadii* lacks NPQ under low or high light intensities.

**Supplementary Figure S3.** NPQ is not induced by high-light or acidifying conditions in *C. ohadii* grown under strict photoautotrophic conditions.

**Supplementary Figure S4.** 1O2 detection with SOSG probe workflow.

## Dive Curated Terms

The following phenotypic, genotypic, and functional terms are of significance to the work described in this paper:

ATP CHEBI: CHEBI:15422

PGR5 Gramene: AT2G05620
PGR5 Araport: AT2G05620
