# Supplementary material for: Processes independent of nonphotochemical quenching protect a high-light-tolerant desert alga from oxidative stress
Source: Plant Physiol. 2024 Nov 9;197(1):kiae608. doi: 10.1093/plphys/kiae608 (PMC11663709; doi:10.1093/plphys/kiae608)
Supplement: kiae608_Supplementary_Data [file kiae608_supplementary_data.zip › Fig S1.pptx]

## Slide 1
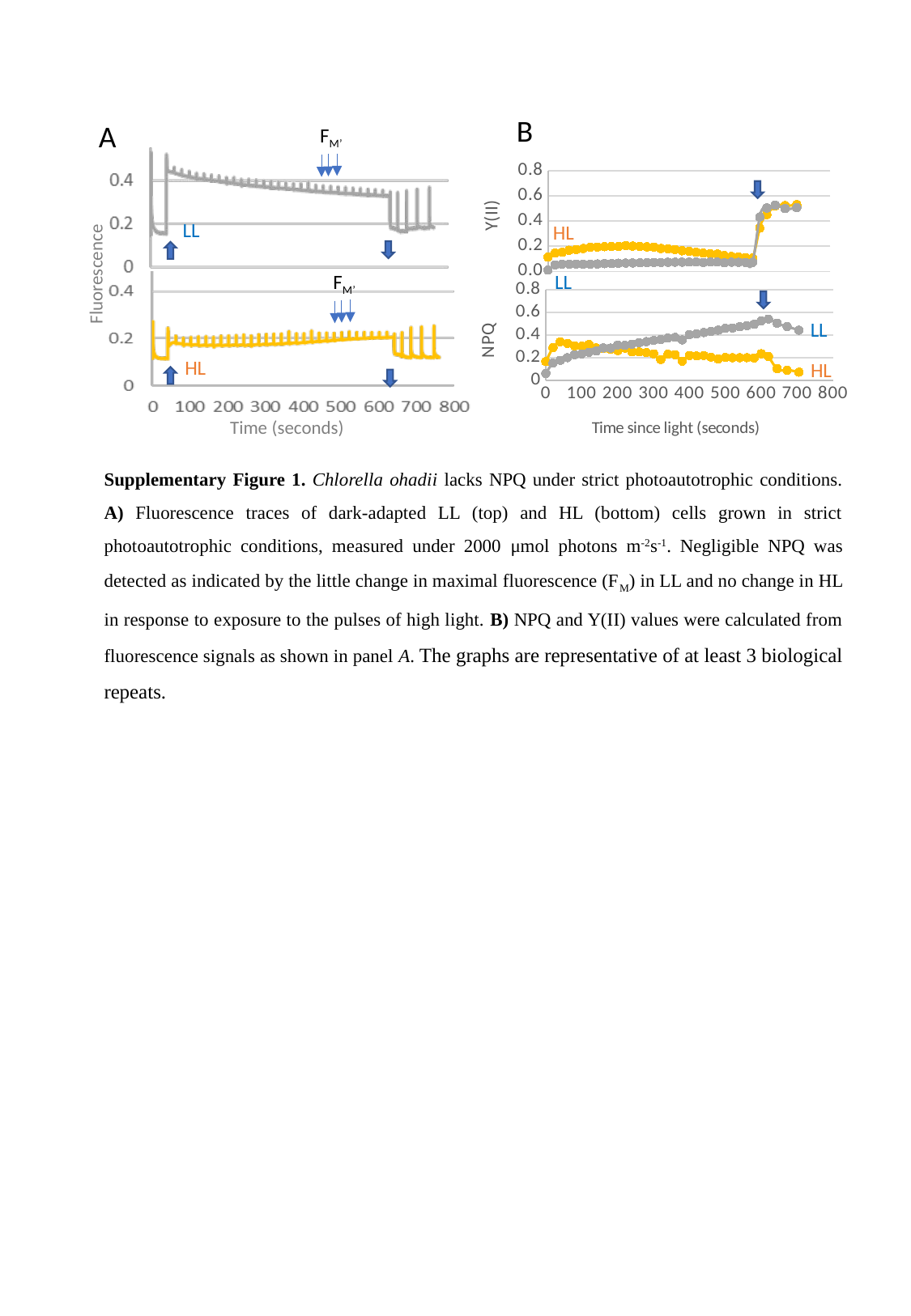

B
A
FM’
### Chart
| Category | | |
|---|---|---|
LL
HL
Fluorescence
FM’
LL
### Chart
| Category | | |
|---|---|---|
LL
HL
HL
Time (seconds)
Supplementary Figure 1. Chlorella ohadii lacks NPQ under strict photoautotrophic conditions. A) Fluorescence traces of dark-adapted LL (top) and HL (bottom) cells grown in strict photoautotrophic conditions, measured under 2000 μmol photons m-2s-1. Negligible NPQ was detected as indicated by the little change in maximal fluorescence (FM) in LL and no change in HL in response to exposure to the pulses of high light. B) NPQ and Y(II) values were calculated from fluorescence signals as shown in panel A. The graphs are representative of at least 3 biological repeats.
