# Supplementary material for: Processes independent of nonphotochemical quenching protect a high-light-tolerant desert alga from oxidative stress
Source: Plant Physiol. 2024 Nov 9;197(1):kiae608. doi: 10.1093/plphys/kiae608 (PMC11663709; doi:10.1093/plphys/kiae608)
Supplement: kiae608_Supplementary_Data [file kiae608_supplementary_data.zip › Fig S2.pptx]

## Slide 1
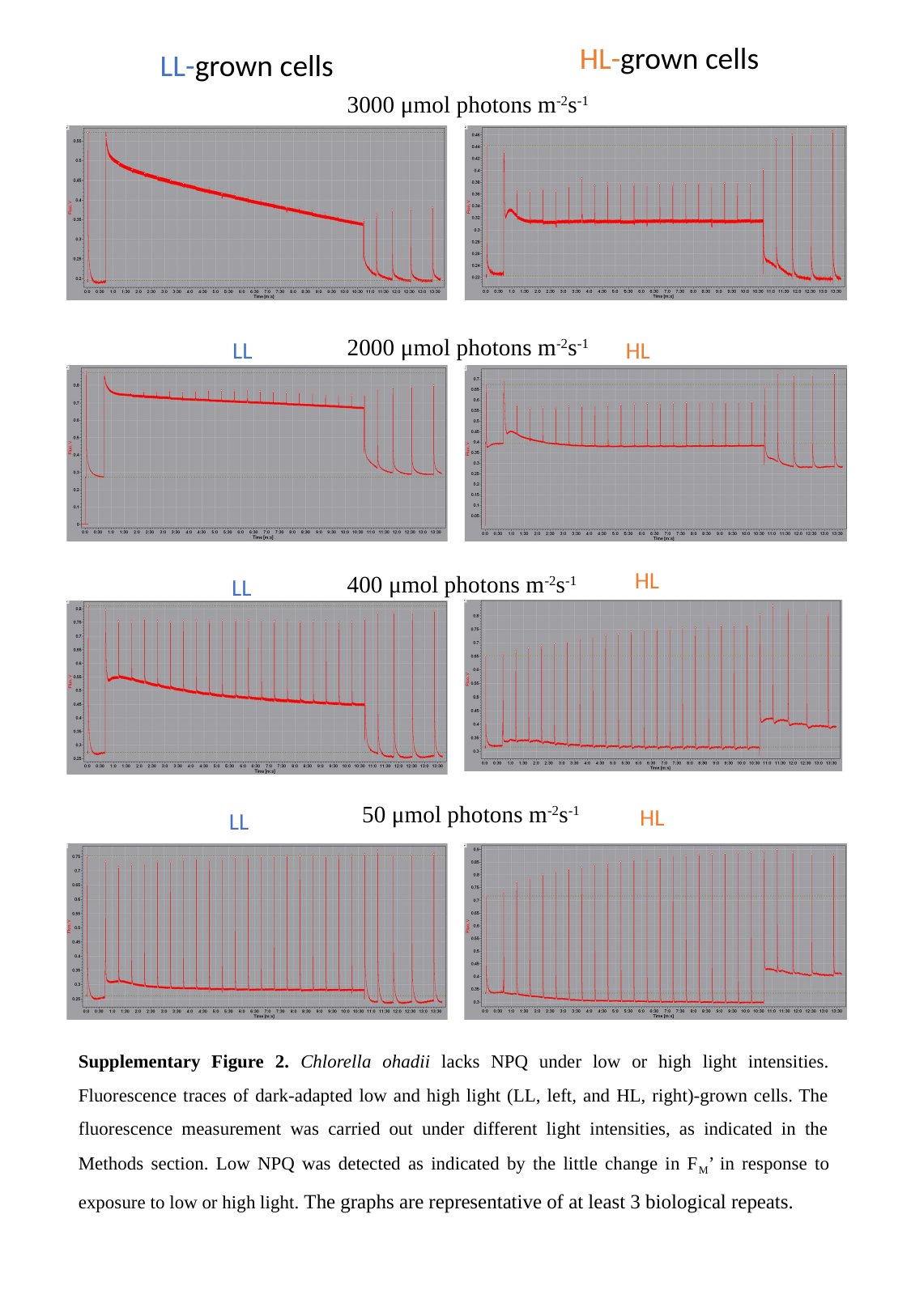

HL-grown cells
LL-grown cells
3000 μmol photons m-2s-1
2000 μmol photons m-2s-1
LL
HL
HL
400 μmol photons m-2s-1
LL
50 μmol photons m-2s-1
HL
LL
Supplementary Figure 2. Chlorella ohadii lacks NPQ under low or high light intensities. Fluorescence traces of dark-adapted low and high light (LL, left, and HL, right)-grown cells. The fluorescence measurement was carried out under different light intensities, as indicated in the Methods section. Low NPQ was detected as indicated by the little change in FM’ in response to exposure to low or high light. The graphs are representative of at least 3 biological repeats.
