# Supplementary material for: Processes independent of nonphotochemical quenching protect a high-light-tolerant desert alga from oxidative stress
Source: Plant Physiol. 2024 Nov 9;197(1):kiae608. doi: 10.1093/plphys/kiae608 (PMC11663709; doi:10.1093/plphys/kiae608)
Supplement: kiae608_Supplementary_Data [file kiae608_supplementary_data.zip › Fig S3.pptx]

## Slide 1
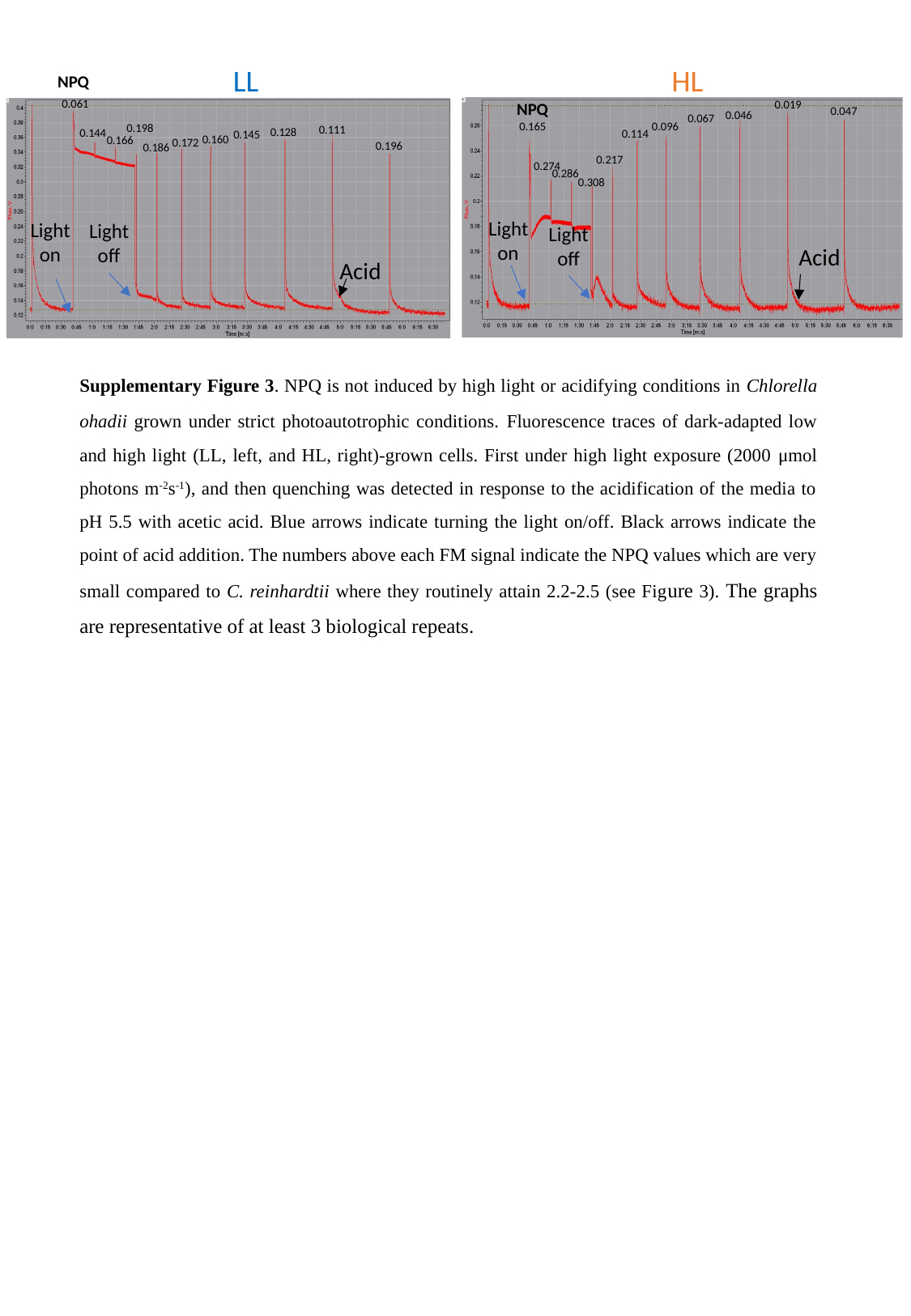

LL
NPQ
 0.061
0.198
0.111
0.128
0.144
0.145
0.160
0.166
0.172
0.196
0.186
Acid
HL
0.019
NPQ
0.165
0.047
0.067
0.096
0.114
0.274
0.286
0.308
Acid
0.046
0.217
Light on
Light on
Light off
Light off
Supplementary Figure 3. NPQ is not induced by high light or acidifying conditions in Chlorella ohadii grown under strict photoautotrophic conditions. Fluorescence traces of dark-adapted low and high light (LL, left, and HL, right)-grown cells. First under high light exposure (2000 μmol photons m-2s-1), and then quenching was detected in response to the acidification of the media to pH 5.5 with acetic acid. Blue arrows indicate turning the light on/off. Black arrows indicate the point of acid addition. The numbers above each FM signal indicate the NPQ values which are very small compared to C. reinhardtii where they routinely attain 2.2-2.5 (see Figure 3). The graphs are representative of at least 3 biological repeats.
