# Supplementary material for: Processes independent of nonphotochemical quenching protect a high-light-tolerant desert alga from oxidative stress
Source: Plant Physiol. 2024 Nov 9;197(1):kiae608. doi: 10.1093/plphys/kiae608 (PMC11663709; doi:10.1093/plphys/kiae608)
Supplement: kiae608_Supplementary_Data [file kiae608_supplementary_data.zip › Fig S4.pptx]

## Slide 1
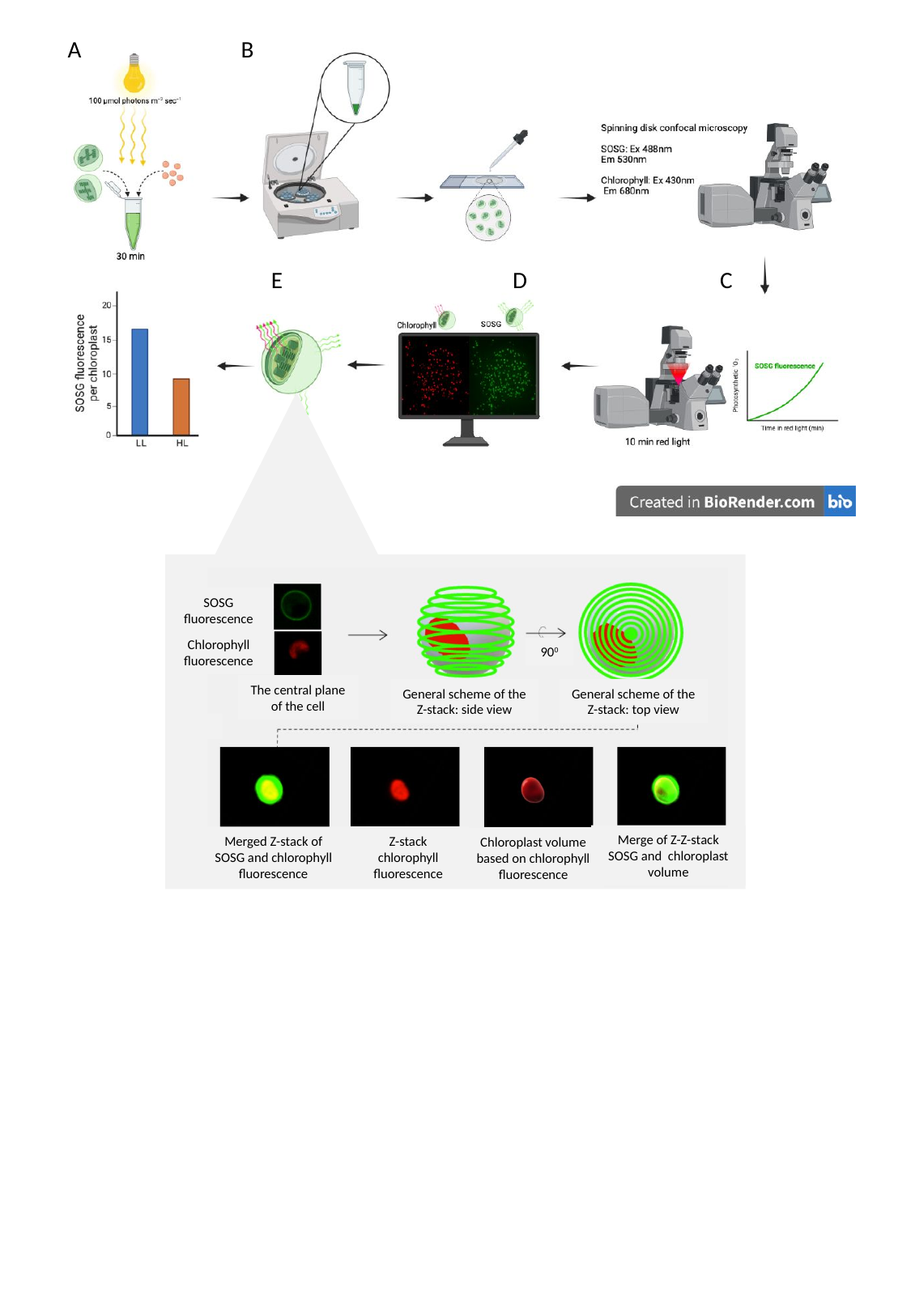

A
B
E
D
C
SOSG fluorescence
Chlorophyll fluorescence
900
The central plane of the cell
General scheme of the Z-stack: side view
General scheme of the Z-stack: top view
Merge of Z-Z-stack SOSG and chloroplast volume
Z-stack chlorophyll fluorescence
Merged Z-stack of SOSG and chlorophyll fluorescence
Chloroplast volume based on chlorophyll fluorescence

## Slide 2
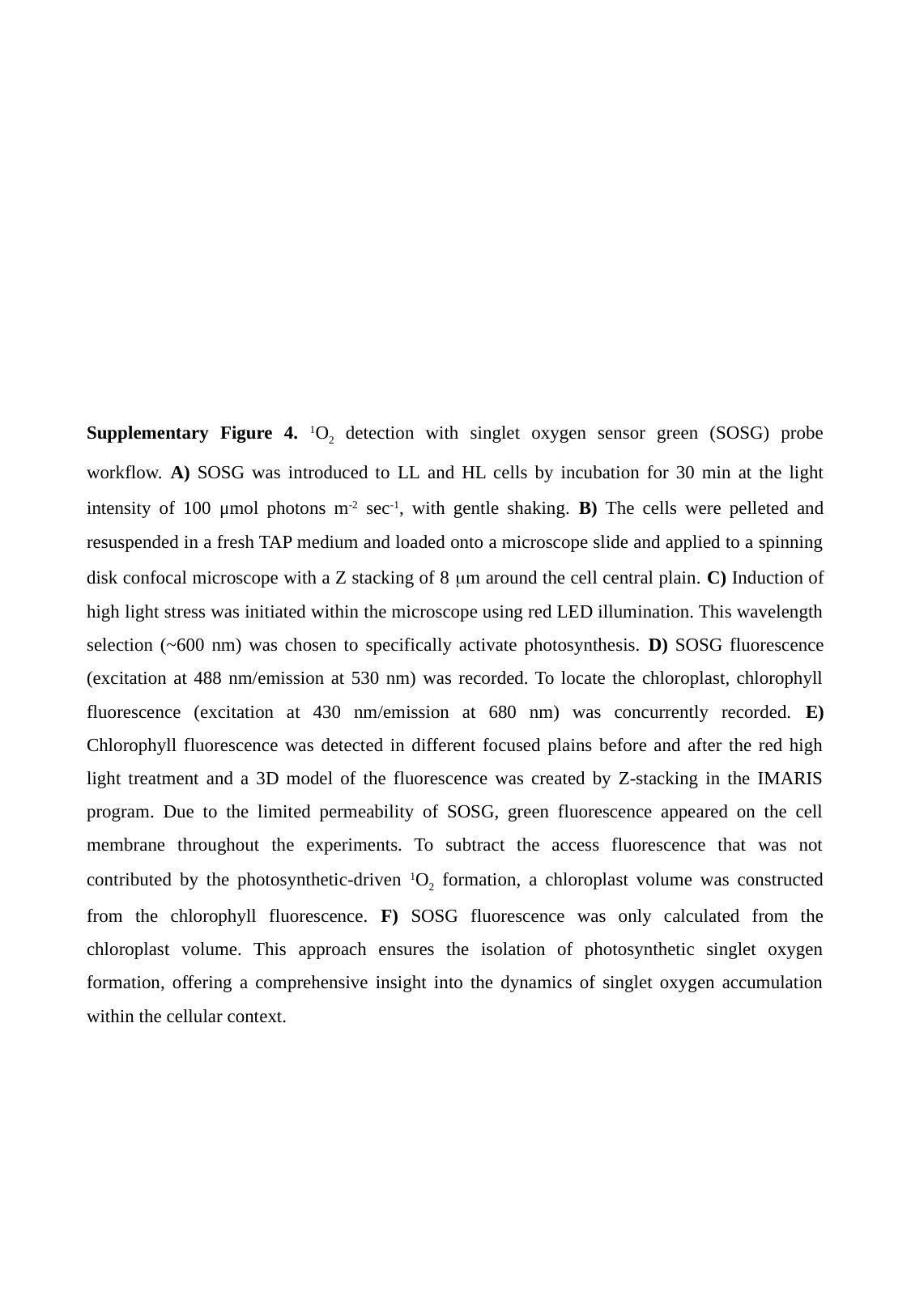

Supplementary Figure 4. 1O2 detection with singlet oxygen sensor green (SOSG) probe workflow. A) SOSG was introduced to LL and HL cells by incubation for 30 min at the light intensity of 100 μmol photons m-2 sec-1, with gentle shaking. B) The cells were pelleted and resuspended in a fresh TAP medium and loaded onto a microscope slide and applied to a spinning disk confocal microscope with a Z stacking of 8 mm around the cell central plain. C) Induction of high light stress was initiated within the microscope using red LED illumination. This wavelength selection (~600 nm) was chosen to specifically activate photosynthesis. D) SOSG fluorescence (excitation at 488 nm/emission at 530 nm) was recorded. To locate the chloroplast, chlorophyll fluorescence (excitation at 430 nm/emission at 680 nm) was concurrently recorded. E) Chlorophyll fluorescence was detected in different focused plains before and after the red high light treatment and a 3D model of the fluorescence was created by Z-stacking in the IMARIS program. Due to the limited permeability of SOSG, green fluorescence appeared on the cell membrane throughout the experiments. To subtract the access fluorescence that was not contributed by the photosynthetic-driven 1O2 formation, a chloroplast volume was constructed from the chlorophyll fluorescence. F) SOSG fluorescence was only calculated from the chloroplast volume. This approach ensures the isolation of photosynthetic singlet oxygen formation, offering a comprehensive insight into the dynamics of singlet oxygen accumulation within the cellular context.
