# Supplementary material for: Processes independent of nonphotochemical quenching protect a high-light-tolerant desert alga from oxidative stress
Source: Plant Physiol. 2024 Nov 9;197(1):kiae608. doi: 10.1093/plphys/kiae608 (PMC11663709; doi:10.1093/plphys/kiae608)
Supplement: kiae608_Supplementary_Data [file kiae608_supplementary_data.zip › Supplementary Data.pdf]

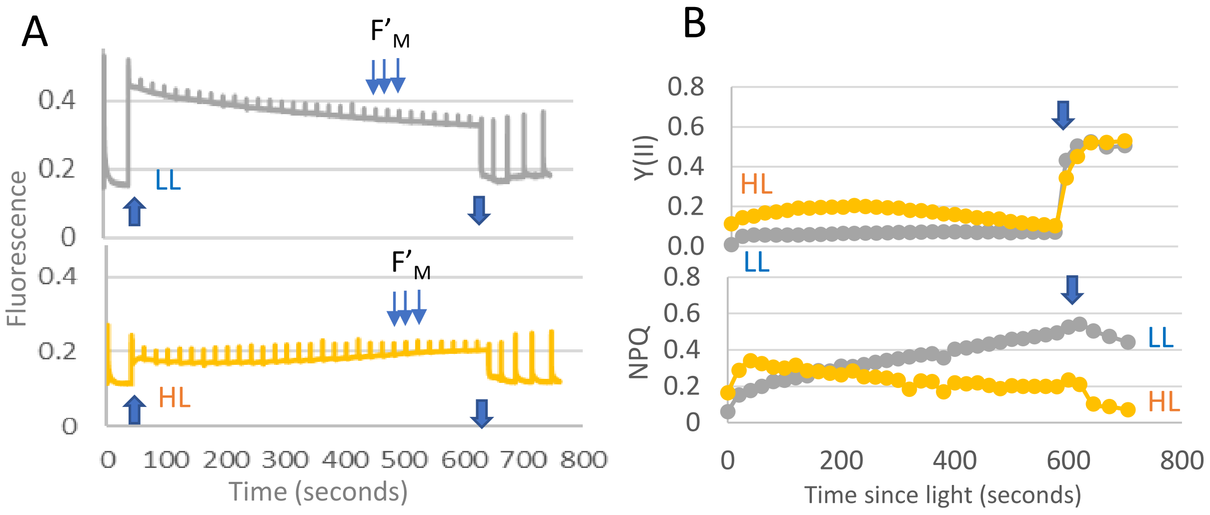


**Supplementary Figure S1.** *Chlorella ohadii* lacks NPQ under strict photoautotrophic conditions. **A)** Fluorescence traces of dark-adapted LL (top) and HL (bottom) cells grown in strict photoautotrophic conditions, measured under 2000 µmol photons $m^{-2}s^{-1}$. Negligible NPQ was detected as indicated by the little change in maximal fluorescence ($F'_M$) in LL and no change in HL in response to exposure to the pulses of high light. Wide blue arrows indicate turning the actinic light on (pointing up) or off (pointing down). Narrow blue arrows indicate $F'_M$ during exposure to actinic light. **B)** NPQ and Y(II) values were calculated from fluorescence signals as shown in panel *A*. The graphs are representative of at least 3 biological repeats. Blue arrows indicate turning the actinic light off.

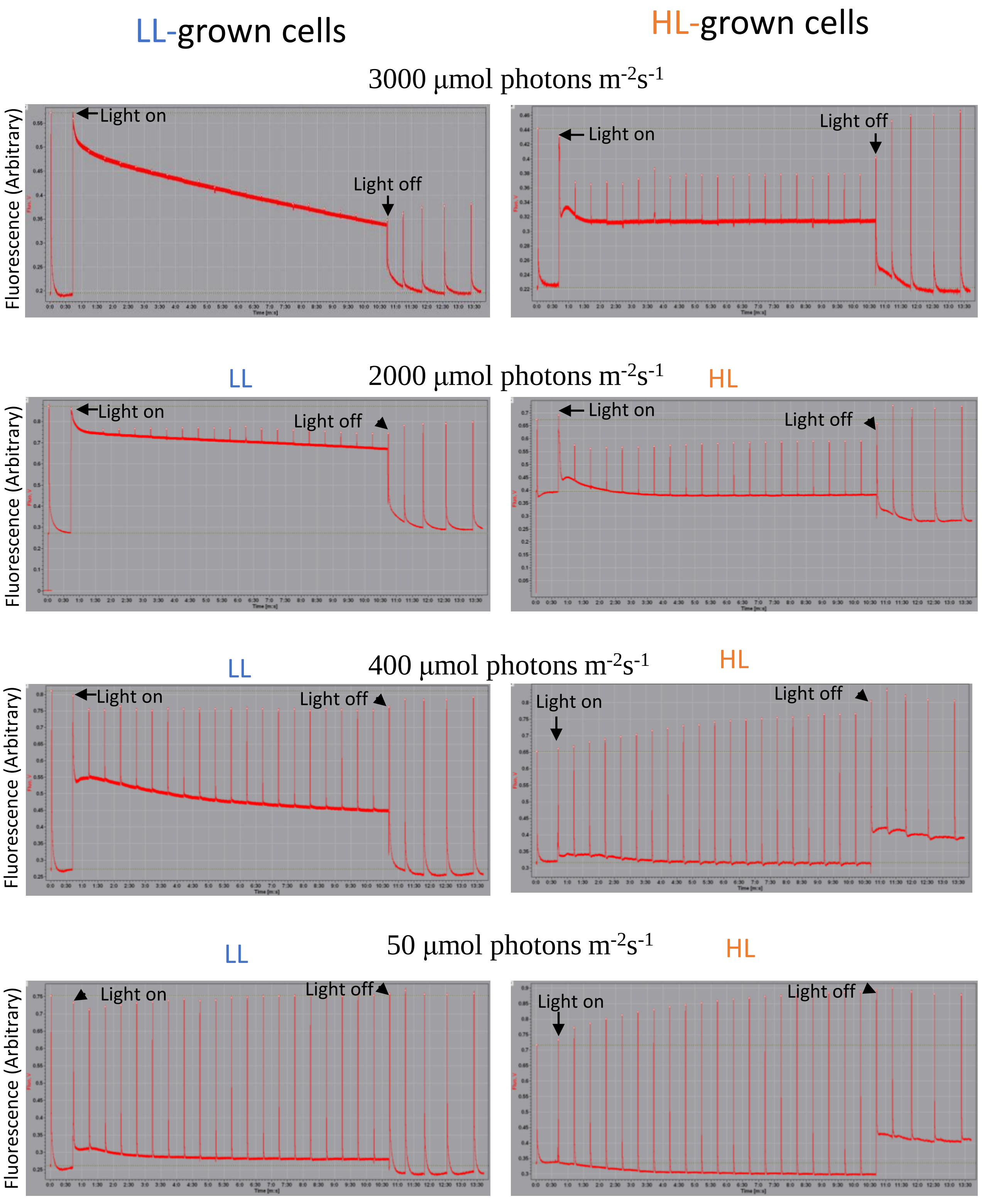


**Supplementary Figure S2.** *Chlorella ohadii* lacks NPQ under low or high light intensities. Fluorescence traces of dark-adapted low and high light (LL, left, and HL, right)-grown cells. The fluorescence measurement was carried out under different light intensities, as indicated in the Methods section. Low NPQ was detected as indicated by the little change in $F_M$' in response to exposure to low or high light. Black arrows indicate turning the actinic light on or off. The graphs are representative of at least 3 biological repeats.

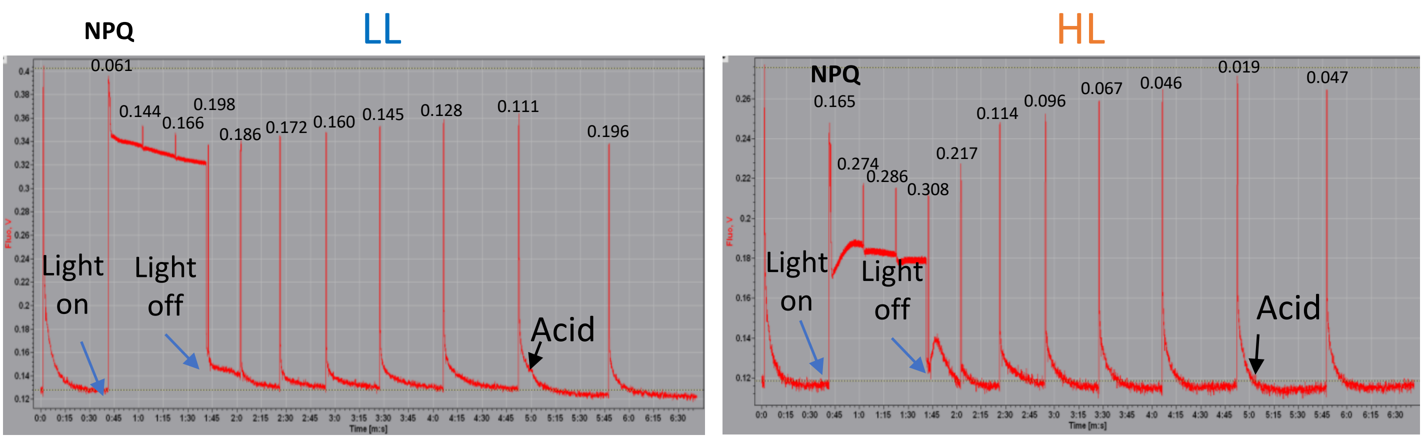


**Supplementary Figure S3**. NPQ is not induced by high light or acidifying conditions in *Chlorella ohadii* grown under strict photoautotrophic conditions. Fluorescence traces of dark-adapted low and high light (LL, left, and HL, right)-grown cells. First under high light exposure (2000 µmol photons $m^{-2}s^{-1}$), and then quenching was detected in response to the acidification of the media to pH 5.5 with acetic acid. Blue arrows indicate turning the light on/off. Black arrows indicate the point of acid addition. The numbers above each FM signal indicate the NPQ values which are very small compared to *C. reinhardtii* where they routinely attain 2.2-2.5 (see Figure 3). The graphs are representative of at least 3 biological repeats.

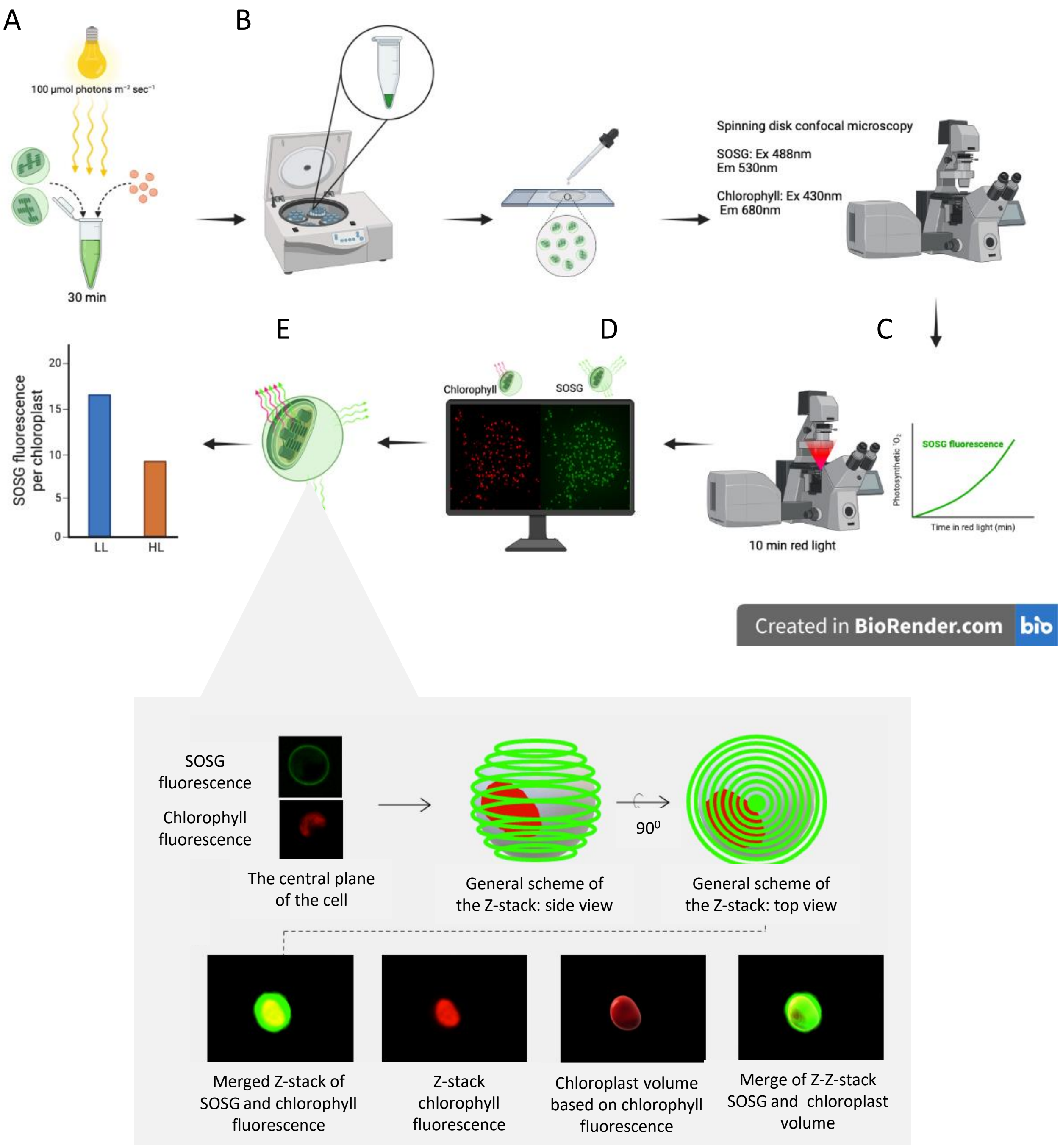
A
B
100 µmol photons m⁻² sec⁻¹
30 min
Spinning disk confocal microscopy
SOSG: Ex 488nm
Em 530nm
Chlorophyll: Ex 430nm
Em 680nm
C
D
E
Chlorophyll
SOSG
10 min red light
SOSG fluorescence
Photosynthetic ¹O₂
Time in red light (min)
SOSG fluorescence per chloroplast
20
15
10
5
0
LL
HL
Created in BioRender.com
SOSG fluorescence
Chlorophyll fluorescence
The central plane of the cell
General scheme of the Z-stack: side view
90⁰
General scheme of the Z-stack: top view
Merged Z-stack of SOSG and chlorophyll fluorescence
Z-stack chlorophyll fluorescence
Chloroplast volume based on chlorophyll fluorescence
Merge of Z-Z-stack SOSG and chloroplast volume

**Supplementary Figure S4.** $^1O_2$ detection with singlet oxygen sensor green (SOSG) probe workflow. **A)** SOSG was introduced to LL and HL cells by incubation for 30 min at the light intensity of 100 µmol photons $m^{-2}$ $sec^{-1}$, with gentle shaking. **B)** The cells were pelleted and resuspended in a fresh TAP medium and loaded onto a microscope slide and applied to a spinning disk confocal microscope with a Z stacking of 8 µm around the cell central plain. **C)** Induction of high light stress was initiated within the microscope using red LED illumination. This wavelength selection (~600 nm) was chosen to specifically activate photosynthesis. **D)** SOSG fluorescence (excitation at 488 nm/emission at 530 nm) was recorded. To locate the chloroplast, chlorophyll fluorescence (excitation at 430 nm/emission at 680 nm) was concurrently recorded. **E)** Chlorophyll fluorescence was detected in different focused plains before and after the red high light treatment and a 3D model of the fluorescence was created by Z-stacking in the IMARIS program. Due to the limited permeability of SOSG, green fluorescence appeared on the cell membrane throughout the experiments. To subtract the access fluorescence that was not contributed by the photosynthetic-driven $^1O_2$ formation, a chloroplast volume was constructed from the chlorophyll fluorescence. **F)** SOSG fluorescence was only calculated from the chloroplast volume. This approach ensures the isolation of photosynthetic singlet oxygen formation, offering a comprehensive insight into the dynamics of singlet oxygen accumulation within the cellular context.
