# Supplementary material for: Processes independent of nonphotochemical quenching protect a high-light-tolerant desert alga from oxidative stress
Source: Plant Physiol. 2024 Nov 9;197(1):kiae608. doi: 10.1093/plphys/kiae608 (PMC11663709; doi:10.1093/plphys/kiae608)
Supplement: kiae608_Supplementary_Data [file pp.02147.2024-s09.pdf › pp.02147.2024-s09.pdf]

Plant Physiology®

A high-light tolerant alga from the desert is protected from oxidative stress by NPQ-independent responses

Guy Levin, Michael Yasmin, Oded Liran, Rawad Hanna, Oded Kleifeld, Guy Horev, Francis-Andre Wollman, Gadi Schuster, and Wojciech Nawrocki

Review Timeline:

Submission Date:	25-Jul-2024
Editorial Decision:	18-Aug-2024
Revision Received:	23-Sep-2024
Editorial Decision:	07-Oct-2024
Revision Received:	10-Oct-2024
Accepted:	17-Oct-2024

18-Aug-2024

Dr. Guy Levin
Technion Israel Institute of Technology
Haifa
Israel

RE: A high-light tolerant alga from the desert is protected from oxidative stress by NPQ-independent responses

Dear Dr. Levin:

We have received reviews of your manuscript, which you may view at the end of this letter. On the basis of the advice received and my own evaluation, I believe that, pending revision, this article may ultimately be acceptable for publication in Plant Physiology.

Two expert scientists have read your submission and provided supportive comments with which I agree. Reviewer 1 requests some clarifications or additions, e.g. reference to a Chlamydomonas mutant with partly similar responses, discussion or inclusion of data on state transition, flavodiiron activity as part of a possible pseudocyclic pathway, and expanded discussion. Reviewer 2 has made many minor comments, but, in addition, this reviewer wishes a more differentiated dealing with reactive oxygen production in the photosynthetic electron transport chain. The clarity of data acquisition and statistics is not really satisfactory. Sometimes you refer to statistics described in Material and Methods. This does not ease reading and understanding the data quality in the figures. A short description of the entire statistics should be provided in the legends. And also in many figures, you should confirm that you have seen this behavior in independent experiments. This has been well done in some figures such as figure 5B - although again you refer to the M&M section, can you name the test here? -, but in other cases you do not indicate how often done and whether the differences are significant (Fig. 4C, D). Why don't you show averaged data in Fig. 2B and 3B.

When you are ready to submit your revision, click the below link and find the red arrow under your Author Tasks heading for this revision submission.

If you want to visit this version of the manuscript, you may click the link below and then click on the folder that reads "Post Decision Manuscripts".

Link Not Available

When uploading the revised version of this article, please be sure to include as complete an answer as possible to the referees' criticisms during the first round of reviews in the "response to reviewers" section.

Plant Physiology considers a revision for up to 60 days after a manuscript has been provisionally accepted; limited extension of this timeframe is at the discretion of the Monitoring Editor. A reminder notice will be sent to you 2 weeks before the deadline, at which time you may choose to contact the editorial office to request an extension; if so, please provide a justification for your request. After the expiration of this deadline, the article would need to be submitted as a resubmission and would be handled as such.

Thank you very much for giving us an opportunity to review this work. I look forward to receiving the next version.

Sincerely,

Editor, Plant Physiology

Want to add this revision deadline to your calendar? Click below!

-- Reviewer comments:

Reviewer #1 (Comments for the Author):

In the submission "A high-light tolerant alga from the desert is protected from oxidative stress by NPQ-independent responses", the team investigate a fascinating alga, Chlorella ohadii, that thrives in the desert. They compare high (2000µE) and low light (50µE) acclimated cells. The authors reveal that unlike in higher plants and many green algae investigated so far that under high light C. ohadii completely breaks down light harvesting complexes (LHC). As such, high-energy state quenching (qE)-type NPQ is non-existent, since this NPQ mechanism involves dissipating energy absorbed by LHC. Importantly, in low light acclimated cells with intact LHC, the team show that qE-type NPQ is still absent. When placed under high light, low light acclimated cells produce more ROS than high light acclimated cells due to excess light absorption and absence of NPQ. Chlorella ohadii does not possess LHC-stress-related (LHCSR)-type proteins that typically contribute to qE in green algae, explaining the absence of qE also in low light acclimated cells. I find the submission interesting and experiments well performed. Some responses of high light acclimated C. ohadii, e.g. light curves and very high electron-transport rates, are reflective of the LHC-truncated Chlamydomonas reinhardtii tla2 mutant (Kirst et al., 2012 PLANT PHYSIOL), and this would be worth mentioning in the text.

The authors have focused on qE-type NPQ, but have not measured state transitions (qT). Although apparently absent in high light acclimated C. ohadii presumably due to absence of LHC, variation in LHC abundance could make C. ohadii an interesting model to measure qT. Have the authors tried measuring response of low light cells with PAM chlorophyll fluorescence to far-red light, or investigated phosphorylation of thylakoid proteins?

Amongst some other interesting observations, the proteomic data shows a 3-fold accumulation of FLV1, but then the authors fail to revisit this later during assessments of alternative electron flows. Therefore, it would also be insightful if the authors assessed flavodiiron (FLV) activity, e.g. with near infra-red absorbance measurement of photosystem I (P700). Presumably with elevated FLV1 levels it is not only cyclic electron flow, but also pseudocyclic electron flow that is upregulated under high light. They should also be considered and added to Figure 6 as a potentially important regulatory mechanism of electron flow, which also prevents ROS production via the Mehler reaction (Pfleger et al 2024; BBA).

In the discussion the authors say on line 335 "C. ohadii provides a remarkable demonstration of what NPQ is not suited for". I would like to see support for this statement from other studies of similar desert-dwelling algae/lichen, since this could be an isolated case rather than a rule.

In my opinion, what is currently missing from the text is how the low and high light treatments used in the study to acclimate the cells correspond with the light intensity cells are in their natural environment. DO they inhabit environments of light intensities, i.e. are the algae on the upper or lower surfaces of sand in the desert?
It is not always apparent in the text whether cells for each experiment were from heterotrophic or photoautotrophic cultures. On occasion in results mention is made of photoautotrophic cells, but only in the methods did I see that cells were often grown heterotrophically. This is important because it affects cell response to contrasting light intensities, including oxidative damage.
On line 420 'MIN' is mentioned as a media but I could not find which media this is exactly.

Reviewer #2 (Comments for the Author):

Review of "A high-light tolerant alga from the desert is protected from oxidative stress by NPQ-independent responses" by Levin et al.

In this current manuscript, Levin et al. demonstrate that the light-tolerant algae Chlorella ohadii uses an NPQ-independent mechanism to avoid ROS production and photo-inhibition. This is accomplished by performing a series of experiments with cells that have acclimated to either "low light" (50 µE) or "high light" (2,000 µE), with the latter mimicking full and direct sunlight that this species regularly is subjected to in its desert habitat. Through a series of physiological assays, the authors measure the differences in photosynthesis, protein accumulation, and ROS accumulation in these two groups of cells. They observe that the "high light" group has reduced ROS accumulation and protein oxidation, but without the activation of NPQ. Instead, these cells have reduced PSII antenna and increased CEF around PSI.

Overall, I find this to be a well written manuscript describing well-conducted experiments. The findings that this extremophile algae uses a potentially novel mechanism to avoid photo-damage under physiological conditions should also be of general interest to the field of photosynthesis and ROS biology fields.

My major concern is how the manuscript oversimplifies the issue of ROS being generated by photosynthesis. The two major types produced, singlet oxygen (1O2) and superoxide (O2-) are made through distinct mechanisms in the chloroplast and cells use different mechanisms to quench them. For instance, the enzymes discussed in the main text that are observed to accumulate under high light conditions, are used to detoxify cells from O2- and hydrogen peroxide (H2O2). There are no known enzymes that quench 1O2. Instead, non-enzymatic mechanisms involving molecules such as carotenoids and tocopherols are used. I think the authors need to address these nuances in their introduction and in how they interpret their results. For instance, O2- and H2O2 detoxification may be occurring through the up-regulation of SOD and APX, respectively. However, what could

be leading to 1O2 quenching? Can the authors measure carotenoids? This could also help test if increased CBR protein is somehow linked to carotenoid levels.

Relatively minor comments

1. Line 42. "molecular players" is vague. Please be more specific.
2. Line 43. In general, I think the term "excess light" should be used instead of "high light." What is high light to one organism may be low light to another. Instead, the term excess light takes a specific organism's photosynthetic capacity into consideration.
3. Line 97. I think it is important to state how long the acclimation timeframe is in the results and in the discussion. This is an important parameter for the experiment. If a shorter time was used (~30 min), very different results could have been obtained.
4. Lines 105-114. How many of the up-regulated proteins are plastid-localized? Is ROS detoxification only occurring in this organelle or is a whole-cell response occurring?
5. Line 133. Could an interpretation of this data be that the "high light" cells have increased protein turnover, which leads to less accumulation of damaged proteins?
6. Line 213. Mutations should be italicized.
7. Line 221. To me, the SOSG experiment seems to only be measuring 1O2 being produced by the confocal laser, and does not necessarily reflect what is being produced under the two light conditions. If the authors agree, this should be stated. Is there a way to measure 1O2 under high light? I imagine this may be technically tricky due to the autoactivation properties of SOSG. Also, the authors are only reporting the amount of SOSG signal in chloroplasts. I would be interested to know if signal was detected in the cytoplasm and if it was different in the two light regimes. One can imagine that some level of 1O2 may leak out of chloroplasts if damaged.
8. Line 234. Please state in the results how superoxide and hydrogen peroxide were measured.
9. Line 256 "The led us to assess..."
10. Line 316. Protein turnover was not measured here, so the authors should be careful in making such statements.
11. Line 354. Please avoid descriptors like "impressive" to describe data.
12. Line 376. As stated above, the reduction of 1O2 cannot be achieved by ROS-scavenging enzymes.
13. Fig .1 legend. This experiment describes acclimation, not adaptation.

Note: **The given line numbers are correct when the changes (visible via the track changes feature) are *NOT* displayed in the edited manuscript.**

Dear Editor and Reviewers,

We thank you very much for your valuable feedback and comments. We found them very helpful and we believe they have helped us to significantly improve our paper. Please find below a point-by-point response to each of the comments:

Editor comments:

Two expert scientists have read your submission and provided supportive comments with which I agree. Reviewer 1 requests some clarifications or additions, e.g. reference to a Chlamydomonas mutant with partly similar responses, discussion or inclusion of data on state transition, flavodiiron activity as part of a possible pseudocyclic pathway, and expanded discussion. Reviewer 2 has made many minor comments, but, in addition, this reviewer wishes for a more differentiated dealing with reactive oxygen production in the photosynthetic electron transport chain.

Answer: We thank you and the reviewers for your positive response and valuable feedback. Please find below a point-by-point response to the comments you and the reviewers raised.

The clarity of data acquisition and statistics is not really satisfactory. Sometimes you refer to statistics described in Material and Methods. This does not ease reading and understanding the data quality in the figures. A short description of the entire statistics should be provided in the legends. And also in many figures, you should confirm that you have seen this behaviour in independent experiments. This has been well done in some figures such as figure 5B - although again you refer to the M&M section, can you name the test here? -, but in other cases, you do not indicate how often it is done and whether the differences are significant (Fig. 4C, D). Why don't you show averaged data in Fig. 2B and 3B.

Answer: Thank you for your important comment. We indeed agree that the clarity of the statistics presentation was lacking. We have made improvements to the legends of all figures to address this issue and we hope they are now satisfactory. We also provide better explanations in the manuscript text for some of these issues.

Reviewer #1 (Comments for the Author):

I find the submission interesting and the experiments well performed. Some responses of high light acclimated *C. ohadii*, e.g. light curves and very high electron-transport rates, are reflective of the LHC-

truncated *Chlamydomonas reinhardtii* tla2 mutant (Kirst et al., 2012 PLANT PHYSIOL), and this would be worth mentioning in the text.

Answer: We thank the reviewer for his encouraging words and valuable comment. We now discuss the similarity to the *tla2* Chlamy mutant and provide the suggested reference. (Lines 388-393)

The authors have focused on qE-type NPQ, but have not measured state transitions (qT). Although apparently absent in high light acclimated *C. ohadii* presumably due to the absence of LHC, variation in LHC abundance could make C. ohadii an interesting model to measure qT. Have the authors tried measuring the response of low light cells with PAM chlorophyll fluorescence to far-red light, or investigated the phosphorylation of thylakoid proteins?

Answer: We thank the reviewer for his comment. We would like to point out that we address this issue in a previous paper (Levin et al., 2023 https://doi.org/10.1111/tpj.16241) We induced state transition with chemicals and analyzed their 77K fluorescence spectra. Our results show very little state transitions in LL-grown cells and no state transitions at all was detected in HL-cells. We also used two approaches to address photosynthetic protein phosphorylation: 1. phospho-immunoblots and 2. proteomics. Our analysis suggested negligible phosphorylation of PSII-LHCII proteins. In all of the experiments, Chlamydomonas was used as positive control to demonstrate active state transitions.

We made it clearer in the text that we have previously tested State transitions and protein phosphorylation and better explain the importance of this observation in light of our new results (qE is lost in addition to qT). (Lines 73, 75, 237-239 and 367)

Amongst some other interesting observations, the proteomic data shows a 3-fold accumulation of FLV1, but then the authors fail to revisit this later during assessments of alternative electron flows. Therefore, it would also be insightful if the authors assessed flavodiiron (FLV)activity, e.g. with near infra-red absorbance measurement of photosystem I (P700). Presumably with elevated FLV1 levels it is not only cyclic electron flow, but also pseudocyclic electron flow that is upregulated under high light. They should also be considered and added to Figure 6 as a potentially important regulatory mechanism of electron flow, which also prevents ROS production via the Mehler reaction (Pfleger et al 2024; BBA).

Answer: We thank the reviewer for pointing out the omission to comment on that phenotype – indeed, we have found an increase in the FLVs abundance. However, our functional data argues against their significant role at the onset of illumination in the HL-grown cells. In the manuscript we do present the P700 measurement, which we agree with the reviewer are important to dissect the roles of alternative electron flows. In these measurements, we observe a strong increase in the time

needed to oxidize P700, at contrast to what would be expected from an increase in the FLVs function. As shown in the report from Chaux et al. (Plant phys., 2017, Fig, 8A, but note that the timescales are different due to the absence of DCMU addition), these enzymes increase the electron flow rate by oxidizing the acceptor side of PSI. Given that our data shows an even longer lag in HL-grown cells despite an upregulation in the FLVs abundance, we ought to conclude that we have underestimated the upregulation of CEF which seems even stronger due to the increased competition with FLVs. We now discuss this briefly in the manuscript (lines 304-308).

In the discussion the authors say on line 335 "*C. ohadii* provides a remarkable demonstration of what NPQ is not suited for". I would like to see support for this statement from other studies of similar desert-dwelling algae/lichen since this could be an isolated case rather than a rule.

Answer: We thank the reviewer for his comment. We added some references from other desert organisms to support our claim. However, as far as we are aware, the relevant data for such organisms is scarce and anecdotal. We agree that our statement is too conclusive and we have softened it, and we now also emphasize the need for more to determine whether the observed NPQ loss is common across species. (Lines 368-373)

In my opinion, what is currently missing from the text is how the low and high light treatments used in the study to acclimate the cells correspond with the light intensity cells are in their natural environment. Do they inhabit environments of light intensities, i.e. are the algae on the upper or lower surfaces of sand in the desert?

Answer: We thank the reviewer for his comment. The algae grow in very thin layers on the sand in what is known as a biological soil crust (BSC), where they are exposed to almost direct sunlight. The depth in/on the surface may vary slightly, however, the algae that are on the uppermost layers is indeed expected to be exposed to light intensities similar, or close to our tested HL conditions (2000 μE) – during the summer. Our low light conditions may represent the light intensity in deeper layers of the BSCs or on the (rare) cloudy winter days. However, the chosen light conditions have as a goal to demonstrate the flexibility of the photosynthetic apparatus in C. ohadii rather than mimic precisely two particular conditions in the desert. In other words, it is an approximation as data about the *exact* light intensity that *C. ohadii* is exposed to in its natural habitat is not available.

To make it clearer we now discuss the relevance of our chosen light intensities to the natural light intensity in *C. ohadii's* habitat. (Lines 105-111)

It is not always apparent in the text whether cells for each experiment were from heterotrophic or photoautotrophic cultures. On occasion in results mention is made of photoautotrophic cells, but only

in the methods did I see that cells were often grown heterotrophically. This is important because it affects cell response to contrasting light intensities, including oxidative damage. On line 420'MIN' is mentioned as a media but I could not find which media this is exactly.

Answer: We thank the reviewer for his comment. All experiments were done under heterotrophic conditions unless stated otherwise, as the cells were grown in these conditions to ensure optimal growth and prevent carbon limitation. Where we determined it was necessary, (NPQ measurements and qE induction by acid) we repeated the experiments under strict photoautotrophic conditions (cell growth in the MIN medium with no reduced carbon source) to demonstrate that similar results are obtained in both growth conditions and refer to these experiments in the text (Lines 212 and 228, Figures S1 and S3 respectively).

We now describe at the beginning of the results section that our cells were grown heterotrophically (Lines 107-108). We also now clearly indicate in the materials and methods section in which experiments photoautotrophic conditions were employed (Line 462).

Reviewer #2 (Comments for the Author):

Overall, I find this to be a well written manuscript describing well-conducted experiments. The findings that this extremophile algae uses a potentially novel mechanism to avoid photo-damage under physiological conditions should also be of general interest to the field of photosynthesis and ROS biology fields.

Answer: We thank the reviewer for his generous words.

My major concern is how the manuscript oversimplifies the issue of ROS being generated by photosynthesis. The two major types produced, singlet oxygen (1O2) and superoxide (O2-) are made through distinct mechanisms in the chloroplast and cells use different mechanisms to quench them. For instance, the enzymes discussed in the main text that are observed to accumulate under high light conditions, are used to detoxify cells from O2- and hydrogen peroxide (H2O2). There are no known enzymes that quench 1O2. Instead, non-enzymatic mechanisms involving molecules such as carotenoids and tocopherols are used. I think the authors need to address these nuances in their introduction and in how they interpret their results. For instance, O2- and H2O2 detoxification may be occurring through the up-regulation of SOD and APX, respectively. However, what could be leading to 1O2 quenching? Can the authors measure carotenoids? This could also help test if increased CBR protein is somehow linked to carotenoid levels.

Answer: We thank the reviewer for this valuable comment. We now distinguish between singlet oxygen and superoxide, and between their quenching mechanisms, throughout the manuscript

including the introduction, results, and discussion. The O2- and H2O2 detoxification can be achieved via the accumulated enzymes (with SOD and GPX appearing to be mostly accumulated). The 1O2 detoxification likely mainly occurs via carotenoids, which we have previously shown to be massively accumulated in HL-grown *C. ohadii* (Levin et al. 2023 https://doi.org/10.1111/tpj.16241). CBR, which is the most accumulated protein in HL cells compared to LL cells, was previously suggested to harbor carotenoids. In *C. ohadii*, we found CBR to co-localize with carotenoids on a sucrose density gradient. Potentially, CBR contributes to the direct scavenging of 1O2 via bound carotenoids. Simultaneously, the bound carotenoids may be used to directly quench excited chlorophylls and thus prevent ROS generation.

This is now better explained and presented throughout the manuscript. Several examples: Lines 43-51, 115-118, 131-134, 148-149 157-163, 419-420, and more.

Relatively minor comments

1. Line 42. "molecular players" is vague. Please be more specific.

Answer: 'Molecular players' was changed to 'Photosynthetic proteins' (Line 42).

2. Line 43. In general, I think the term "excess light" should be used instead of "high light." What is high light to one organism may be lowlight to another. Instead, the term excess light takes a specific organism's photosynthetic capacity into consideration.

Answer: We have changed 'High light' with 'excess light' in the proposed line and several other places where it was appropriate. However, we prefer to keep using high light (HL) in some cases as we use it as an abbreviation for our growth conditions (i.e HL-grown cells) and we think it helps the reader to follow what has been done. It is also consistent with our previous works (Levin et al 2021, 2023, 2024).

3. Line 97. I think it is important to state how long the acclimation timeframe is in the results and in the discussion. This is an important parameter for the experiment. If a shorter time was used (~30 min), very different results could have been obtained.

Answer: We added the timeframe of the acclimation process in the results and discussion as suggested. (Lines 106 and 344).

4. Lines 105-114. How many of the up-regulated proteins are plastid-localized? Is ROS detoxification only occurring in this organelle or is a whole-cell response occurring?

Answer: Unfortunately, the annotations of the Chlorella proteome are not fully specified. Thus, in most cases, we are unable to distinguish between chloroplast or other localized protein homologs

based on mass spectrometry identifications. We do not have sufficient data to confirm the subcellular localization of the identified proteins.

5. Line 133. Could an interpretation of this data be that the "high light" cells have increased protein turnover, which leads to less accumulation of damaged proteins?

Answer: We thank the reviewer for this comment. Yes, it could be a possibility. We added a sentence about this scenario in the discussion (Lines 424-425)

6. Line 213. Mutations should be italicized.

Answer: We thank the reviewer for pointing this out. We fixed it.

7. Line 221. To me, the SOSG experiment seems to only be measuring 1O2 being produced by the confocal laser, and does not necessarily reflect what is being produced under the two light conditions. If the authors agree, this should be stated. Is there a way to measure 1O2under high light? I imagine this may be technically tricky due to the autoactivation properties of SOSG. Also, the authors are only reporting the amount of SOSG signal in chloroplasts. I would be interested to know if signal was detected in the cytoplasm and if it was different in the two light regimes. One can imagine that some level of 1O2 may leak out of chloroplasts if damaged.

Answer: We thank the reviewer for his comment. Indeed, this experiment aims to measure the 1O2 formation, induced by high red light, omitted by the confocal microscope. We made slight modifications to the text (Line 249) and changed the title of this section to be clearer: 'HL-grown cells accumulate less damaging ROS than LL-grown cells under high red-light illumination' (Line 243). High red light (~600 nm) was chosen specifically to avoid autofluorescence by SOSG (excitation at 488 nm). This is also now explained in the text (Lines 250-251).

As seen in Figure 5A, the majority of the recorded fluorescence is confined to the chloroplast or the cell wall/membrane. We specifically measured the fluorescence from the chloroplast to isolate photosynthetically induced 1O2. The SOSG was obtained mainly from a membrane that engulfs several cells together and much less from the cytoplasm. However, in low-light grown cells and following 10 min of high red-light illumination, the signal obtained from the chloroplasts was significantly stronger than those obtained from the other parts of the cells (Figure 5A 10). Our method was not sensitive enough to accurately differentiate between the SOSG signal from the cytoplasm of LL or HL-grown cells. We suspect that 1O2 "leak" from the chloroplasts may take place only after a very prolonged damage that results in a loss of chloroplast membrane continuity.

8. Line 234. Please state in the results how superoxide and hydrogen peroxide were measured.

Answer: We thank the reviewer for his comment. In addition to the description in the materials and methods section (Lines 533-542), we have now described the measuring method both in the results section (Lines 260-263) and in the Figure 4 legend.

9. Line 256 "The led us to assess..."

Answer: We thank the reviewer for pointing this out. We fixed it.

10. Line 316. Protein turnover was not measured here, so the authors should be careful in making such statements.

Answer: We have removed the statement about the turnover.

11. Line 354. Please avoid descriptors like "impressive" to describe data.

Answer: The word ‘impressive’ was removed.

12. Line 376. As stated above, the reduction of 1O2 cannot be achieved by ROS-scavenging enzymes.

Answer: The sentence was rewritten to clarify that O2- quenching can be achieved via the accumulated enzymes, while 1O2 quenching can be achieved via the accumulated carotenoids. (Lines 419-420 and 148-149)

13. Fig .1 legend. This experiment describes acclimation, not adaptation.

Answer: ‘Adaptation’ was changed to ‘Acclimation’.

We wish to thank the reviewers once again and hope that the manuscript it now ready for publication.

Best regards,

Guy Levin and Wojciech Nawrocki.

07-Oct-2024

Dr. Guy Levin
Technion Israel Institute of Technology
Haifa
Israel

RE: A high-light tolerant alga from the desert is protected from oxidative stress by NPQ-independent responses

Dear Dr. Guy Levin:

We are pleased to accept your manuscript for publication in the Plant Physiology. This acceptance is contingent on revision based on the comments of our reviewers. In particular, please consider the following:

Thanks for carefully revising your manuscript. Both reviewers and I are happy with the new version, there is a handful of real minor points. But they probably should be addressed prior to final acceptance.

Please highlight all changes and include a detailed annotation to changes to the text, with line numbers, and noting your responses to the comments.

To submit your revised manuscript, click:

Link Not Available

If you cannot return the revised manuscript within 8 weeks of receipt, please let us know. Otherwise, we will assume that you have elected not to revise the manuscript and are withdrawing it.

Thank you for allowing us to review your work. We look forward to hearing from you soon.

Sincerely,

Editor, Plant Physiology

-- Reviewer comments:
Reviewer #1 (Comments for the Author):

I thank the authors for their detail responses to my comments. I am happy with the changes they have made and would only have a couple of minor suggestions they could consider in their final submission.

1) Superoxide is often abbreviated with the radical indicated as a superscript dot (e.g. shortcut key: Alt+0149)

2) in their summary Figure (Fig.7) it is strange to have all ROS coming from PSII when the Mehler reaction that produces superoxide is mostly considered deriving from PSI. Furthermore, SOD & GPX appear to be in the thylakoid membrane. Is this intentional? Perhaps some ROS (in A, B and C) and these two enzymes (in C) could be moved towards the stroma near PSI.

Reviewer #2 (Comments for the Author):

Based on comments from myself and reviewer #1, the authors have made significant changes to the text and the manuscript has been improved. Overall, I feel that my comments were adequately addressed, and the authors made appropriate changes to the text. I thank the authors for taking our suggestions seriously.

A few more minor points that should be considered before publication:

1. Line 250. The authors should cite a source for using red light to avoid SOSG autofluorescence (e.g., Prasad et al. 2018). https://pubmed.ncbi.nlm.nih.gov/30209276/
2. Line 259. Do the authors mean to mention superoxide or singlet oxygen here?
3. Line 347. Add reference for the observation of carotenoid accumulation.
4. Discussion section. One may hypothesize that without NPQ, C. ohadii may be sensitive to light fluctuations. Are there data to support this?

<u>Note:</u> The given line numbers are correct when the changes (visible via the track changes feature) are *NOT* displayed in the edited manuscript.

Dear Editor and Reviewers,

We thank you very much for your valuable feedback and comments. We found them very helpful and we believe they have helped us to significantly improve our paper. Please find below a point-by-point response to each of the comments:

Reviewer #1 (Comments for the Author):

I thank the authors for their detail responses to my comments. I am happy with the changes they have made and would only have a couple of minor suggestions they could consider in their final submission.

1) Superoxide is often abbreviated with the radical indicated as a superscript dot (e.g. shortcut key: Alt+0149)

Answer: We thank the reviewer for pointing this out. Superoxide is now abbreviated correctly throughout the manuscript: $O_2^{\bullet-}$

2) in their summary Figure (Fig.7) it is strange to have all ROS coming from PSII when the Mehler reaction that produces superoxide is mostly considered deriving from PSI. Furthermore, SOD & GPX appear to be in the thylakoid membrane. Is this intentional? Perhaps some ROS (in A, B and C) and these two enzymes (in C) could be moved towards the stroma near PSI.

Answer: We thank the reviewer for this valuable comment. We have fixed Figure 7 according to the suggestions.

Reviewer #2 (Comments for the Author):

Based on comments from myself and reviewer #1, the authors have made significant changes to the text and the manuscript has been improved. Overall, I feel that my comments were adequately addressed, and the authors made appropriate changes to the text. I thank the authors for taking our suggestions seriously.

A few more minor points that should be considered before publication:

1. Line 250. The authors should cite a source for using red light to avoid SOSG autofluorescence (e.g., Prasad et al. 2018). https://pubmed.ncbi.nlm.nih.gov/30209276/

Answer: We thank the reviewer for this comment. We now point to the suggested reference in the text and the reference list. Lines 251 and 785-787

2. Line 259. Do the authors mean to mention superoxide or singlet oxygen here?

Answer: We mean superoxide. We agree this was not clear and thank the reviewer for pointing this out. We added a reference to Fig. 4D to make it clearer. Line 260.

3. Line 347. Add reference for the observation of carotenoid accumulation.

Answer: We thank the reviewer for his comment we added the relevant references: (Levin et al. 2021, 2023) Line 347

4. Discussion section. One may hypothesize that without NPQ, C. ohadii may be sensitive to light fluctuations. Are there data to support this?

Answer: We thank the reviewer for raising this possibility, and we completely agree. Sadly, we do not currently have data to support this notion. However, we intend to include these experiments in our next manuscript. We now propose this scenario in the discussion. Lines 379-380

We wish to thank the reviewers once again and hope that the manuscript it now ready for publication.

Best regards,

Guy Levin and Wojciech Nawrocki.

17-Oct-2024
Dr. Guy Levin
Technion Israel Institute of Technology
Haifa
Israel

MSID: PP2024-RA-02147R2
MS TITLE: A high-light tolerant alga from the desert is protected from oxidative stress by NPQ-independent responses

Dear Dr. Guy Levin:

I am pleased to inform you that your revised manuscript "A high-light tolerant alga from the desert is protected from oxidative stress by NPQ-independent responses" has been accepted for publication in Plant Physiology. Your manuscript will be checked for consistency with journal formatting and image standards by one of our Science Editors, who will contact you shortly. They may have a number of queries and suggestions for you. Please keep in mind that they work to increase the online visibility of your article and its attraction for readers from the widest of backgrounds. You will have the opportunity to review any revisions, and I hope you will work constructively with them to get the most out of your research. You will also have the opportunity to review all changes when you receive the author proofs for your article.

Once validated for publication, your article will be sent to a Science Editor for a final review of your manuscript and source files. After your final files are uploaded, your article will appear in the next available issue and the online Preview version of your article will be posted after your signed Copyright and Checklist forms and final source files are received.

Please be sure to include the following in your manuscript file:
Harvard style citations
List of all author contributions and funding information at the beginning of manuscript after the titles and author list.
List all supplemental materials associated with the article, with titles, at the end of the ms, before the references and acknowledgments section.
Complete Figure Legends for all figures appearing in the article listed before the Literature Cited.

LICENSE INFORMATION Shortly after your paper is sent to production, you will receive an email from our publisher's author support team, **SciPris**, with information on article processing charges, as well as the types of Open Access license you would be able to purchase. The accepted version of your manuscript will not appear on our Advance Articles page until the license is selected and signed, so please look out for an email from SciPris. For more information see the Article Publication Fees section of our author guidelines: https://academic.oup.com/plcell/pages/General-Instructions

Finally, we encourage your submission of artwork for the journal cover. If there is an image or illustration related to your paper that you would like to have considered, please email it in .pdf or .tif format to clowe@aspb.org. An image title, brief, 2-3 sentence description of the image, and the name of the person credited for the image are also encouraged. If your image is selected to appear on the online cover of the journal, you will be contacted with the scheduled issue date and further information.

Thank you and congratulations on your Plant Physiology paper!

Sincerely,

Editor, Plant Physiology

=====
FIRST AUTHOR PROFILES
=====

We recognize the hard work that goes into being First Author of a Plant Physiology paper by publishing a "First Author Feature" for each First Author alongside your paper.

If the first author(s) would like to submit information for a feature on Facebook and Twitter, fill out the form by clicking on the link below:

Plant Physiology First Author Feature Form: https://forms.office.com/r/ZBCGZfhm8F
If you cannot access the form through the link above, please contact Rachel at rbelsky@aspb.org.

To upload an image of yourself to accompany your profile, please put your photo in the correct journal folder at the link below.
You must label your image: Author First Name Last Name Article Title.

First Author Profile Image Dropbox: https://www.dropbox.com/scl/fo/xz029p7qjr2jml2rp2kmq/h?dl=0&rlkey=6fkxcaqmxx35ap152b2osy1fz

=====
IMPORTANT REMINDER: PEER REVIEW REPORTS
=====

If you opted to publish a peer review report along with your article during the original submission process, it will be prepared by the editorial staff and publicly posted with your manuscript, inside the zip file that contains any other supplemental material. As a reminder, the peer review report is a public record of all comments from editors and reviewers, as well as your prior responses, as you received them in the decision letters for each draft of your manuscript. If you agreed to publish this report and have changed your mind, or are not sure if you selected this option, please contact the editorial office as soon as possible before signing the license agreement from our publisher.

-- Reviewer comments:
